# Bispecific Antibody PD-L1 x CD3 Boosts the Anti-Tumor Potency of the Expanded Vγ2Vδ2 T Cells

**DOI:** 10.3389/fimmu.2021.654080

**Published:** 2021-05-10

**Authors:** Rui Yang, Susu Shen, Cheng Gong, Xin Wang, Fang Luo, Fengyan Luo, Yang Lei, Zili Wang, Shasha Xu, Qian Ni, Yan Xue, Zhen Fu, Liang Zeng, Lijuan Fang, Yongxiang Yan, Jing Zhang, Lu Gan, Jizu Yi, Pengfei Zhou

**Affiliations:** ^1^ Research and Development Department, Wuhan YZY Biopharma Co., Ltd, Wuhan, China; ^2^ National Engineering Research Center for Nanomedicine, College of Life Science and Technology, Huazhong University of Science and Technology, Wuhan, China

**Keywords:** [CD3xPD-L1], Vγ2Vδ2 T cells, adoptive transfer, immunotherapy, NSCLC

## Abstract

Vγ2Vδ2 T cell-based immunotherapy has benefited some patients in clinical trials, but the overall efficacy is low for solid tumor patients. In this study, a bispecific antibody against both PD-L1 and CD3 (PD-L1 x CD3), Y111, could efficiently bridge T cells and PD-L1 expressing tumor cells. The Y111 prompted fresh CD8+ T cell-mediated lysis of H358 cells, but spared this effect on the fresh Vδ2+ T cells enriched from the same donors, which suggested that Y111 could bypass the anti-tumor capacity of the fresh Vγ2Vδ2 T cells. As the adoptive transfer of the expanded Vγ2Vδ2 T cells was approved to be safe and well-tolerated in clinical trials, we hypothesized that the combination of the expanded Vγ2Vδ2 T cells with the Y111 would provide an alternative approach of immunotherapy. Y111 induced the activation of the expanded Vγ2Vδ2 T cells in a dose-dependent fashion in the presence of PD-L1 positive tumor cells. Moreover, Y111 increased the cytotoxicity of the expanded Vγ2Vδ2 T cells against various NSCLC-derived tumor cell lines with the releases of granzyme B, IFNγ, and TNFα *in vitro*. Meanwhile, the adoptive transferred Vγ2Vδ2 T cells together with the Y111 inhibited the growth of the established xenografts in NPG mice. Taken together, our data suggested a clinical potential for the adoptive transferring the Vγ2Vδ2 T cells with the Y111 to treat PD-L1 positive solid tumors.

## Introduction

Vγ2Vδ2 T cells, accounting for about 90% of total γδ T cells in the peripheral bloodstreams of healthy adults, appear to be a fast-acting and non-conventional T-cell population that contributes to both innate and adaptive immune responses to microbial infections and cancers (1). Due to their unique biological functions, Vγ2Vδ2 T cells have been widely used for adoptive cell immunotherapy in clinical trials to treat a broad range of cancer patients who have been resistant to the standard therapies (2). In the past decades, the phase I/II clinical trials demonstrated that the adoptive Vγ2Vδ2 T cell-based therapy was safe, but showed limited efficacy (3). The poor infiltration of the transfused Vγ2Vδ2 T cells into the tumor sites and the anti-tumor activities of Vγ2Vδ2 T cells impaired in the tumor microenvironment may cause the failure of the current therapy (4, 5).

There is an unmet need for the development of novel strategies to improve the therapeutic efficiency of the current Vγ2Vδ2 T cell-based immunotherapy (6). Over three decades ago, Ferrini et al. initially proposed the concept that bispecific antibodies (bsAbs) targeting the γδ TCR and a folate binding protein enhanced the cytotoxic activity of the γδ T cells against human ovarian carcinoma cells (7). Several studies exploited the synergic effects of bsAbs and the Vγ2Vδ2 T cells on fighting tumors in recent years. The combination of bispecific antibodies, (Her2 x CD3) or (Her2 x Vγ2) (8, 9), together with the transferred Vγ2Vδ2 T cells in the presence of IL2, achieved a delay in the growth of pancreatic ductal adenocarcinoma tumor in murine models (10). Another bispecific VHH construct (namely 7D12-5GS-6H4), targeting epidermal growth factor receptor (EGFR) and Vδ2-TCR, was also reported to activate Vγ2Vδ2 T cells (11), and to prolong significantly the survival time of xenograft bearing mice in the presence of the transfused Vγ2Vδ2 T cells with the repeated injections of IL2 (12). Moreover, a recent study demonstrated that the combination of anti-Tim3 mAb, T-cell redirecting bispecific antibody MT110 (EpCAM x CD3), and IL2 could further enhance the anti-tumor effects of the transfused Vγ2Vδ2 T cells in tumor-bearing nude mice (13). However, these bispecific molecules were either originally from mice, which raised the risks of the immunogenicity in human beings, or in a form of VHH structure, which could have a short half-life time in the blood (14). Thus, an IgG-like bispecific antibody would display better pharmacokinetics comparing to those antibody fragments. Although these studies showed that the γδ TCR-based bispecific antibodies displayed modest activities of tumor growth inhibitions with the co-administration of IL2 (7–13), these approaches seemed less attractive than the exploring of CD3-targeting bsAbs. We hypothesized that a tumor associated antigen and CD3-targeting bispecific antibody, rather than targeting to only γδ TCR, would enhance the anti-tumor effects of the transfused Vγ2Vδ2 T cells even without administration of phosphoantiens and IL2 into the animals.

Lung cancer is still the leading cause of the deaths of cancer patients worldwide (15). The clinical response rates to the current first or second-line treatment of non-small cell lung cancer (NSCLC) patients, which accounts for approximately 85% of the total lung cancers, are still unsatisfying (16, 17). The adoptive transfer of Vγ2Vδ2 T cells could reduce the growth of NSCLC cell line-derived xenografts and prolong the survival of tumor-bearing mice (18, 19). Yet, this immunotherapy failed in its efficacy evaluation of clinical trials during the past decades (20–22). Meanwhile, the landscape-changing “Magacurve” for advanced NSCLC showed the therapeutic successes of PD1/PD-L1 blockade (23), even though the monotherapy of anti-PD1/PD-L1 mAb resulted in positive response of only ~ 15-30% of NSCLC patients (24). Hence, a combination strategy of the Vγ2Vδ2 T cells-based adoptive transfer therapy together with PD-L1-targeted therapy is worth to be explored for the NSCLC treatment.

In this study, we designed a novel IgG-like bispecific antibody Y111, targeting both PD-L1 and CD3, on the format of Y-body^®^ in which the anti-PD-L1 half antibody maintains its binding affinity to the PD-L1-positive tumor cells while the anti-CD3 scFv may reduce its binding affinity to the T cells (25, 26). Y111 could bridge the T cells and PD-L1 expressing tumor cells, and prompted fresh CD8+ T cell-mediated lysis of H358 cells but spared this effect on the fresh γδ T cells enriched from the same donors, which suggested that Y111 could bypass the anti-tumor capacity of the fresh Vγ2Vδ2 T cells. We then found that Y111 could selectively trigger the activation of the expanded and purified Vγ2Vδ2 T cells dependent on the presence of PD-L1-positive tumor cells. Furthermore, Y111 enhanced the cytotoxicity of Vγ2Vδ2 T cells against various NSCLC cell lines with the secretion of IFNγ, TNFa, and Granzyme B. Furthermore, the combination of Y111 and transfused Vγ2Vδ2 T cells displayed effective inhibitory effects on the growth of the established xenograft in immunodeficient NPG mice. Taken together, our data demonstrated a new strategy for potentially efficient Vγ2Vδ2 T cell-based immunotherapy for NSCLC and other types of cancers.

## Materials and Methods

### Expression and Purification of Bispecific Antibody

**Figure 1:**
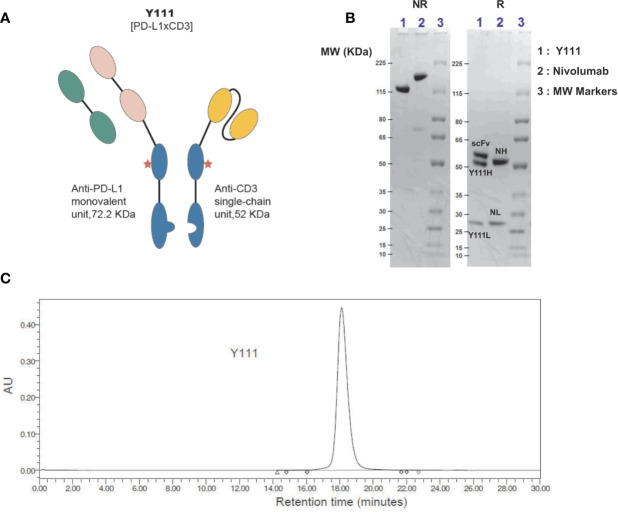
Generation and purification of Y111, a bispecific antibody targeting both CD3 and PD-L1. **(A)** Schematic diagram of bispecific antibody Y111, which consists of a monovalent unit adapted from Tecentriq, a monoclonal antibody targeting PD-L1, and a single-chain variable fragment (scFv) from 2A5 (27), a monoclonal antibody for CD3 activation. The red asterisk indicates N297Q mutation for precluding Fc receptor-mediated crosslinking. **(B)** SDS-PAGE analysis of the purified Y111 under non-reducing (NR, left) and reducing (R, right) conditions. Nivolumab is a monoclonal antibody used as a control. Molecular weight (MW) is indicated in KDa. The MW of Y111 is a little smaller than the monoclonal antibody Nivolumab as indicated from NR gel. There are 3 and 2 bands for Y111and Nivolumab in reducing gel respectively as expected. Please note that the nominal MW of Y111H is 48.850 KDa, Y111L is 23.365 KDa, scFv is 52.057 KDa, and intact Y111 is 124.272 KDa as predicted by their protein sequences. The predicted nominal MW of Nivolumab is 143.597 KDa, the predicted MW of heavy (NH) and light (NL) chain of Nivolumab is 48.422 KDa and 23.359 KDa, respectively. **(C)** Size-exclusion chromatograms of Y111 purified by Protein-A and ion-exchange chromatography. The purity for this Y111 sample is 99.63%.

The Y111 is a recombinant anti-PD-L1 and anti-CD3 (PD-L1 x CD3) bispecific antibody (**Figure 1A**) generated from the CHO cell expression system. The anti-PD-L1 monovalent unit was from the drug bank website (https://go.drugbank.com/drugs/DB11595). The anti-PD-L1 sequence was reversely translated into the DNA sequence, and the anti-CD3 single-chain DNA sequence was reversely translated from the protein sequences of anti-CD3 monoclonal antibody 2A5 (27). These coding gene sequences were synthesized, inserted into the pEASY-T1 vector (Transgene, Beijing, China), and verified by sequencing the entire vectors by Huada Gene (Wuhan, China). The control molecule, CD3 Isotype, targeting both CD3 and fluorescein [derived from Clone 4-4-20 (28)] was similarly constructed (**Supplementary Figure 1**). Subsequently, these expression vectors were transfected into the CHO cells (Invitrogen, Carsbad, USA) using Fecto PRO Reagent (Ployplus, New York, USA) according to the manufacturer’s protocols. After culturing for 7-days, the supernatant was collected and purified serially by Sepharose Fast Flow protein A affinity chromatography column (GE, Milwaukee, USA), Fab Affinity KBP Agarose High Flow Resin (ACROBio systems, Newark, USA), and SP cation exchanged chromatography column (GE, Milwaukee, USA).

### Cancer Cell Lines

Four human NSCLC cell lines, including NCI-H1975 (human adenocarcinoma epithelial cell line, CRL-5908), NCI-H358 (human lung bronchioalveolar carcinoma cell line, CRL-5807), A549 (human adenocarcinoma epithelial cell line, CRL-185), and NCI-H1299 (human NSCLC metastatic cell line, CRL-5803) were purchased from ATCC. Cells were cultured in RPMI 1640 medium (Gibco, New York, USA) supplemented with 10% FBS (ExCell, Clearwater, USA) except for A549, which was cultured in F-12K medium (Gibco, New York, USA) supplemented with 10% FBS. Before culture, the viability and density of cells were determined by the Vi-Cell counter (Beckman Coulter, Indianapolis, USA). All cell lines in use were routinely tested to make sure free of Mycoplasma infection using a 16s-based PCR kit (Vazyme, Nanjing, China), and new cultures were established monthly from frozen stocks as described previously (29).

### Cell Binding and Co-Binding Assays

Cells were incubated in the presence of serially diluted antibodies for 1 hour at room temperature. Subsequently, the cells were washed twice in PBS buffer (PBS+2%FBS+ 2 mM EDTA) and stained for 25 minutes with PE-conjugated anti-human IgG Fc antibody (HP6017, Biolegend, San Diego, USA) diluted in 1:100 into PBS buffer. The bound antibodies were measured using flow cytometry.

To determine the cell-to-cell association mediated by Y111, CFSE-stained H1975 cells were co-cultured with PKH26-labeled Jurkat cells at a ratio of 1:1 with specified concentrations of the Y111 or CD3 Isotype for 1-hour in a 96-well-plate. The samples were measured with a FACSelesta instrument (BD, San Jose, USA) and analyzed with FlowJo software (BD, San Jose, USA). Co-binding% of two cells mediated by bispecific antibodies was indicated as the percentages of both CFSE and PKH26 double-positive cells among the total cells.

### *Ex Vivo* Expansion of PBMCs and Purification of Vγ2Vδ2 T Cells and Other T Cell Subsets

Human peripheral blood mononuclear cells (PBMCs) were first isolated from the fresh blood of randomized healthy donors (LDEBIO, Guangzhou, China) by density gradient centrifugation using Ficoll-Hypaque PLUS (GE, Milwaukee, USA). The purified PBMCs were frozen in liquid nitrogen to mimic the clinic situation in which the frozen PBMCs was usually utilized as the starting point for evaluating the anti-cancer efficiency of the Vγ2Vδ2 T cells. After quick thawing, the cell numbers were counted using AO/PI after staining with Cellometer K2 Fluorescent Cell Viability Counter (Nexcelom Bioscience, Lawrence, USA), and the PBMCs were cultured in RPMI 1640 medium supplemented with 10% FBS, 2.5 μM Zoledronic Acid (Sigma Aldrich, Darmstadt, Germany), and 1000 IU/mL IL2 (Sihuan Pharma, Beijing, China) at 2×10^6^ cells/mL seeded in 6-well-plate as described (30). Every 3 days, half the volume of the culture media was removed and replaced with fresh cell-culture media containing 1000 IU/mL IL2. During days 12-14, Vγ2Vδ2 T cells were purified from the expanded PMBCs by negative selection using the TCR γ/δ + T Cell Isolation Kit (Miltenyi Biotech, Teterow, Germany). The Vγ2Vδ2 T cells purity was assessed by flow cytometry, and the purified (>96%) Vγ2Vδ2 T cells were further cultured in RPMI 1640 medium supplemented with 10% FBS overnight for rest. Then, these Vγ2Vδ2 T cells were used for functional analyses by *in vitro* assays and *in vivo* anti-tumor studies (**Supplementary Figure 2**). In some assays, the T cell subsets were purified from freshly-collected PBMC using the respective negative isolation kits (Miltenyi Biotech, Teterow, Germany) according to the manufacturer’s instructions.

### Intracellular Cytokine Staining (ICS) for T Cell Functional Evaluation

Flow cytometry was performed as described in the previous reports (31, 32). H1975 cells were firstly plated in a 24-wells plate. On the next day, expanded and negatively enriched Vγ2Vδ2 T cells were added into each of the wells with doses of Y111 or CD3 Isotype together with BV510-anti-CD107a (H4A3, Biolegend, San Diego, USA) and BFA (Golgi Plug, BD, San Jose, USA). After co-cultured for 6 hours, the cells were stained with Zombie Fixable Viability Kit (Biolegend, San Diego, USA), followed by incubation with APC-anti-CD3 (SP34-2, BD, San Jose, USA), PE-anti-Vδ2 (B6, Biolegend, San Diego, USA) for 20 min at room temperature in dark. The cells were permeabilized for 30 min at 4 degrees (Cytofix/Cytoperm, BD, San Jose, USA). After wash, the cells were incubated fixation buffer with BV650-anti-IFNγ (4S.B3; Biolegend, San Diego, USA), BV421-anti-TNFα (Mab11, Biolegend, San Diego, USA) for 30 min at room temperature in dark. Then cells were washed and collected by a BD FACSelesta flow cytometry.

### *In Vitro* Tumor Cell Killing Assay

On the first day of the cytotoxicity assay, 2X10^4^ CFSE-labeled target cells were seeded and co-cultured with the enriched- and expanded- Vγ2Vδ2 T cells at an E: T ratio of 1:1, or with the T cell subsets at 1:10 with various doses of indicated antibodies. The cells were incubated at 37°C for 12 h in a humidified CO_2_ incubator. Flow cytometry was used to determine antibody-induced cytotoxic activity-mediated by Vγ2Vδ2 T cells. The percentages of CFSE and PI double-positive cells among the total of target cells (CFSE+) were defined as “Cytotoxicity %”.

### Cytometric Bead Array Method

To measure the cytokines released from Vγ2Vδ2 T cells, the supernatants were harvested from the samples co-cultured with the T cells and tumor cells. Flex Set kits (BD, San Jose, USA) were used to measure the human IFNγ, TNFα, and Granzyme B according to the manufacturer’s instructions. To determine the production of cytokines induced by the antibodies, the raw values were subtracted from the values of E+T groups in the absence of the tested antibodies.

### *In Vivo* Mice Tumor Model Analysis

Female Nonobese diabetic/severe combined immunodeficiency mice (NOD. Cg-Prkdc^scid^ IL2rg^tm1Vst^/Vst, NPG) were obtained from the VITALSTAR (Beijing, China) at ages of 6-8 weeks and housed in the central laboratory in Hubei Province Food and Drug Safety Evaluation Center. 5 x10^^6^ H1975 cells were injected *s.c.* into NPG mice for xenotransplantation on Day 0. On Day 15 when tumor volumes reached about 220 mm^3^, mice were randomly divided into four groups (n = 7 per group). On Day 17, the grouped mice were injected *i.v.* with 1 x10^^6^ purified Vγ2Vδ2 T cells with 1 mg/kg or 4 mg/kg Y111 or PBS as the control. This injection was repeated on Day 20, 24, and 27 (twice a week for 2 weeks).

For each treatment, the purified Vγ2Vδ2 T cells displayed the mature phenotype of the T cells indicated by that the IL2 treatment increased the expressions of CD86, CD69, and HLA-DR (**Supplementary Figure 2**). Tumor volumes were measured with a digital caliper three times a week and calculated using the formula: Tumor Volume (mm^3^) = (a x b^2^)/2, where “a” is the longitudinal length and “b” is the transverse width.

### IHC Analysis

To assess the infiltration and accumulation of transferred Vγ2Vδ2 T cells *in vivo*, mice were sacrificed on Day 39. The tumor tissues were immediately removed, cut into small pieces, and embedded in 4% paraformaldehyde for fixation. Then these tumor pieces were sectioned, stained staining with a rabbit-anti-human CD3 antibody (SP7, Abcam, Cambridge, USA), and examined on a Nikon microscope (Tokyo, Japan). Positive cells were counted in five randomly selected microscopic fields (magnification 20X) and supplied for further quantification analysis.

### Statistical Analyses

Statistical analyses were performed with Prism 6.0 (GraphPad, San Diego, USA) and data were shown as mean± SEM. Non-linear regression methods were applied for analyses of cell binding, co-binding, activation, and cell-based killing activities, and the results were plotted as “Dose-Response Curves”. *P* values were assessed by student’s t-test, nonparametric Mann–Whitney U test, one-way or two-way ANOVA, and Dunnett test or Tukey multiple comparisons as appropriate. *P* values <0.05 were considered significant.

## Results

### Characterization of Y111

Y111 (PD-L1 x CD3), a both PD-L1- and CD3-targeting bispecific antibody, that redirected T cells to attack PD-L1-expressing cancer cells, was designed under the Y-body^®^ platform (25, 26). Y111 consisted of a Fab structure targeting PD-L1, a single-chain variable fragment (scFv) targeting CD3 originated from a monoclonal antibody 2A5 (27) for activating T cells, and a modified Fc region (**Figure 1A**) from human IgG1. The Fc region of Y111 was engineered with the mutations for both “Knob-into-Hole” match for the favorable formation of the heterodimer between the heavy chains and the single chain, and the deficiency of ADCC activity (25). The molecular weight of the Y111 generated from CHO expression was verified by non-reduced and reduced SDS-PAGE analyses (**Figure 1B**). As expected, under reducing conditions the three bands in the gel demonstrated the three chains of Y111, i.e., heavy chain (Y111H: ~ 52 kDa), light chain (Y111L: ~ 28 kDa), and single-chain (scFv: ~ 57 kDa) (**Figure 1B**), while a monoclonal antibody Nivolumab displayed two bands consisting of the heavy (NH) and light (NL) chains (**Figure 1B**). The purity of the Y111 was determined by size-exclusion chromatograms-HPLC (SEC-HPLC) to be > 99% (**Figure 1C**).

### Binding Properties of Y111

**Figure 2:**
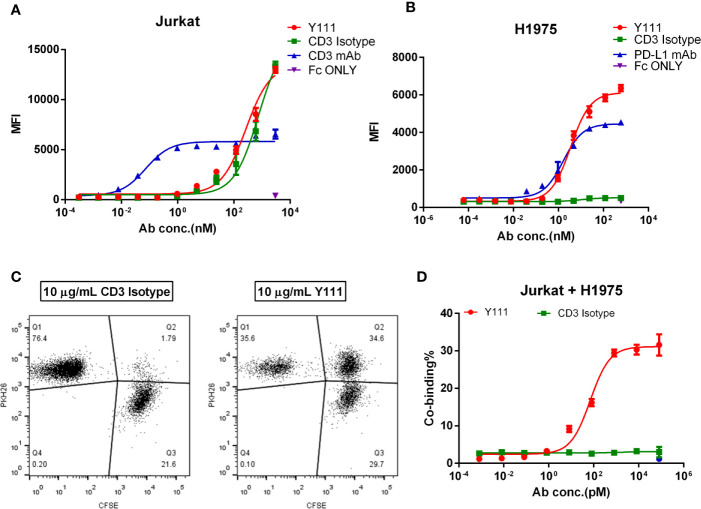
Cell binding activities of Y111. **(A, B)** The binding affinity of Y111 to the CD3 expressed on the Jurkat cells **(A)**, and the PD-L1 expressed on the H1975 cells **(B)**. Flow cytometry was used to assess the geometric mean fluorescence (MFI) of the PE channel, and data were analyzed using the “One Site-Specific binding” method through the least-squares fitting. Plotted dots were the means ± SEM of the triplicate wells from one of three independent experiments. **(C, D)** Y111 bridged the tumor cell and T cells in a dose-dependent manner. CFSE-stained H1975 cells were co-cultured with PKH26-labeled Jurkat cells with a dose of Y111 or CD3 Isotype for 1hour. Co-binding% was indicated as percentages of the CFSE and PKH26 double-positive cells (Q2) among cells. Representative co-binding dot plots were shown in **(C)**, a nonlinear regression depicting dose-dependent-association modulated by Y111was shown in **(D)** Data in **(D)** were represented as mean ± SEM pooled from four independent experiments, and were analyzed using the “log (agonist) vs. response (three parameters)” method through an ordinary fitting. Y111, a bispecific antibody targeting CD3 and PD-L1; CD3 Isotype, a control bispecific antibody targeting CD3 and fluorescein; CD3 mAb and PD-L1 mAb, the parental monoclonal antibody targeting CD3 and PD-L1; Fc only, adding the PE-hFc only.

We assessed the affinity of Y111 at the anti-CD3 moiety on Jurkat cells by flow cytometry. With the structural change to sFv from Fab, it was not surprising that the affinity of Y111 was 360-folds lower than that of 2A5 (the parental CD3 mAb of Y111) to Jurkat cells, with the dissociation constants (Kd) of Y111 and 2A5 binding to the Jurkat cells being 711.4 nM and 1.96 nM, respectively (**Figure 2A**), which were consistent with previous reports (14, 25). The K_D_ values of the Y111 bispecific antibody and its parental PD-L1 mAb binding to H1975 cells were 0.84 nM and 0.21 nM, respectively (**Figure 2B**). The results demonstrated that the tumor cell-based affinity of Y111 to PD-L1 was equivalent to its parental mAb.

CD3-targeting bispecific antibody mediating T cells recruitment to cancer cells is considered to be its critical mechanism of action (MOA) (14). We, therefore, investigated whether Y111 could bridge T cells to tumor cells through its dual binding arms. To this end, Jurkat cells stained with CFSE were incubated with H1975 cells labeled with PKH26 for 1 hour, then the proportion of double-positive cells was measured to represent the bridging activity of Y111 (25). In the presence of the CD3 Isotype (fluorescein x CD3) at 10 μg/mL, the double-positive cell population was 1.79% (**Figure 2C**). In the presence of Y111 at the same concentration the double-positive cell population was 34.6% (**Figure 2C**), suggesting that the Y111 significantly bridges the T cell and tumor cell. This function of Y111 in inducing the tumor cell to T-cell association displayed a dose-dependent manner with EC_50_ ~ 72.1 pM, while the CD3 Isotype control was unable to induce this cell-to-cell association (**Figure 2D**). Taken together, these results demonstrated the unique binding activities of Y111 by the anti-PD-L1 moiety to the tumor cells and by the anti-CD3 moiety to the T cells.

### Y111 Failed to Enhance the Cytotoxicity of the Fresh γδT Cells

**Figure 3:**
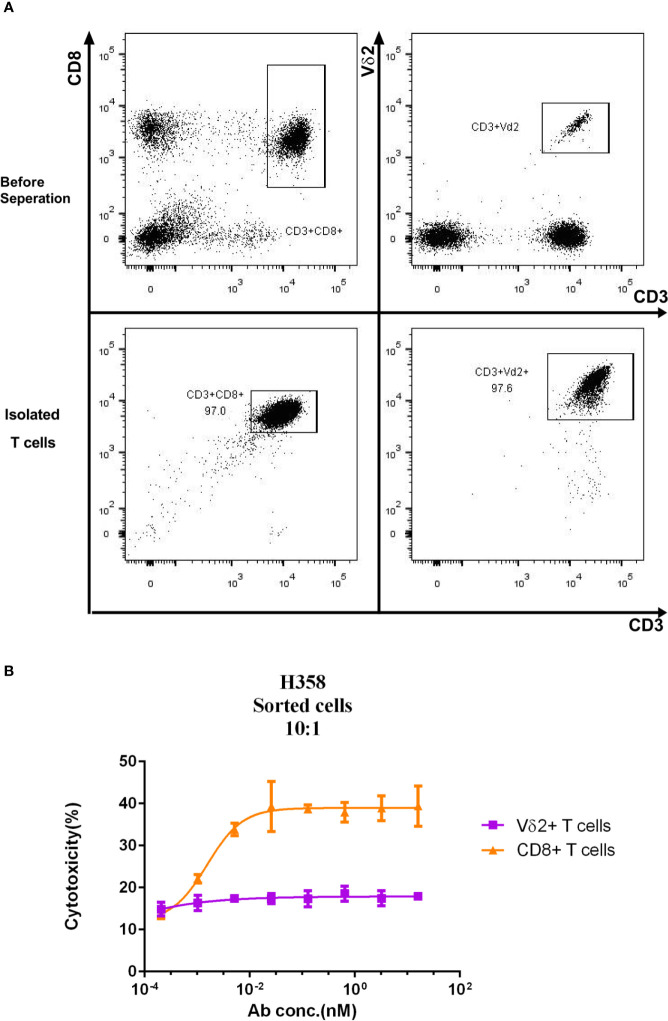
Differential cytotoxicity of fresh CD8+ and Vδ2+ T cells induced by Y111. **(A)** Representative plots show the purity of CD8+, and Vδ2+ T cells enriched from PBMC by negatively magnetic beads separation. **(B)** The purified T-cell subsets from PBMC were co-cultured with CFSE-stained H358 in the presence of serial dilutions of the Y111 for the indicated time, then the proportions of killed target cells (PI+CFSE+) were plotted along with antibody concentration. The dots shown were from 3 independent experiments with T cells obtained from 5 healthy subjects. Data were analyzed using the “log (agonist) vs. response (three parameters)” method through an ordinary fitting.

As the crosslinking of PD-L1 positive target cells with T cells mediated by the Y111 bispecific antibody was expected to cause the effector T-cell–dependent lysis of the target cells (14), we checked whether Y111 redirected the fresh T cells to kill PD-L1 positive tumor cells. To this end, two T-cell subsets including CD8+ and Vδ2+ T cells were negatively isolated from the same PBMCs samples, and co-cultured individually with H358 cells in a ratio of 1:10 (E: T) in the presence of Y111 (**Figure 3A**). Interestingly, we did not observe an elevated effect of Y111 on the cytotoxicity of the fresh Vδ2 T cells, but Y111 increased the effects of CD8+ T cells on lysing the H358 cells in a Y111 dose-dependent fashion (**Figure 3B**). This finding of the difference between the two T-cell subsets was consistent with a previous study using a bispecific antibody targeting Her2 and CD3. These data showed that Y111 prompted the lysis of H358 cells mediated by the fresh CD8+ T cells but spared this effect on the fresh Vδ2 T cells enriched from the same donors, which suggested that Y111 could bypass the anti-tumor capacity of the fresh Vγ2Vδ2 T cells.

### The Activation of the Expanded and Purified Vγ2Vδ2 T Cells by Y111 Was Dependent on the Presence of PD-L1 Expressing Tumor Cells

**Figure 4:**
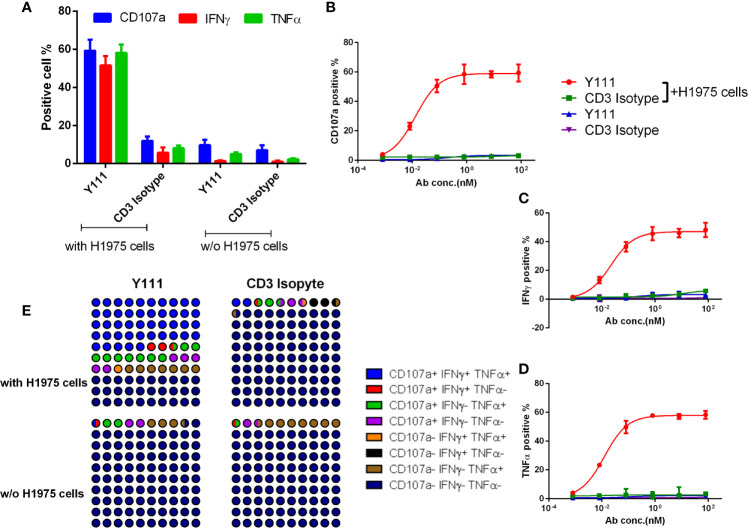
Y111 selectively triggered the cytokine production and degranulation of Vγ2Vδ2 T cells in the presence of target tumor cells. **(A-D)** Y111 efficiently enhanced the activation of Vγ2Vδ2 T cells to produce IFNγ, TNFα, and up-regulate CD107a in tumor cell-dependent fashion. Vγ2Vδ2 T cells were stimulated by Y111 or CD3 Isotype (1 μg/mL of each) **(A)**, and indicated concentration ranges of Y111 or CD3 Isotype **(B–D)** in the presence/absence of H1975 cells in a 1:1 ratio for 6 hours. **(E)** Part of the whole graph shown the co-expression signatures of Vγ2Vδ2 T cells treated by Y111 or CD3 Isotype (1 μg/mL of each) in the presence or absence of H1975 cells. After gating cytokine-positive population (**Supplementary Figure 4**), the boolean analysis was utilized to determine the percentages of multi-functional effector subsets of Vγ2Vδ2 T cells. Bar graph data shown in **(A)** were represented as means ± SEM pooled from three experiments involving 9 healthy donors, dots in **(B–D)** were the means of these individual donors. The data in **(B)** were analyzed using the “log (agonist) vs. response (three parameters)” plot through an ordinary fitting.

As the adoptive transfer of the expanded and purified Vγ2Vδ2 T cells has been shown a safe and well-tolerated therapy (20–22), we tested the concept of the combination of the purified Vγ2Vδ2 T cells with Y111 in the following study. Firstly, we investigated whether Y111 could bridge the expanded Vγ2Vδ2 T cells and tumor cells. To this end, we measured the Y111-mediated co-binding to the tumor cells and Vγ2Vδ2 T cells and found that the Y111 efficiently prompted the double-positive population in the co-culture system with the two types of cells (**Supplementary Figure 3**). Next, the purified Vγ2Vδ2 T cells (the purity and quality of Vγ2Vδ2 T cells were shown in **Supplementary Figure 2**) were cultured with/without tumor cells in the presence of the Y111 in a serial concentrations for 6 hours. We then measured the cell surface expression of CD107a to assess the degranulation of cytotoxic molecules (33), and the intracellular expression of IFNγ and TNFα (34). With the stimulation of both Y111 and tumor cells, a higher proportion of Vγ2Vδ2 T cells displayed potent effector functions and degranulation at 1 μg/mL (~ 8.05 nM), which was not the case for CD3 Isotype (**Figure 4A**, **Supplementary Figure 4**). Furthermore, the considerably unregulated expression of TNFα, IFNγ, and CD107a was aborted in the absence of tumor cells even under the stimulation by Y111 (**Figure 4A**). These data indicated that the activation of Vγ2Vδ2 T cells was controlled jointly by both Y111 and tumor cells. Moreover, this specific activation was in an Y111 dose-depended manner (**Figures 4B–D**). Multifunctional Vγ2Vδ2 T cells have been reported to play central roles in controlling intracellular bacterial infection and killing transformed tumor cells (1, 35). Indeed, we found the co-stimulation of Y111 and H1975 cells induced larger percentages of effector cells to produce multiple cytokines simultaneously (**Figure 4E**). At last, we also observed a dose-depended increase of these multifunctional Vγ2Vδ2 T cells after co-incubation of both the Y111 and tumor cells (**Supplementary Figure 5**). Taken together, these data demonstrated that the efficient activation of Vγ2Vδ2 T cells was dependent on the simultaneous binding of the Y111 to both Vγ2Vδ2 T cells and PD-L1 positive tumor cells.

### Y111 Increased the Killing of PD-L1-Positive NSCLC Cell Lines Mediated by the Expanded and Purified Vγ2Vδ2 T Cells

**Figure 5:**
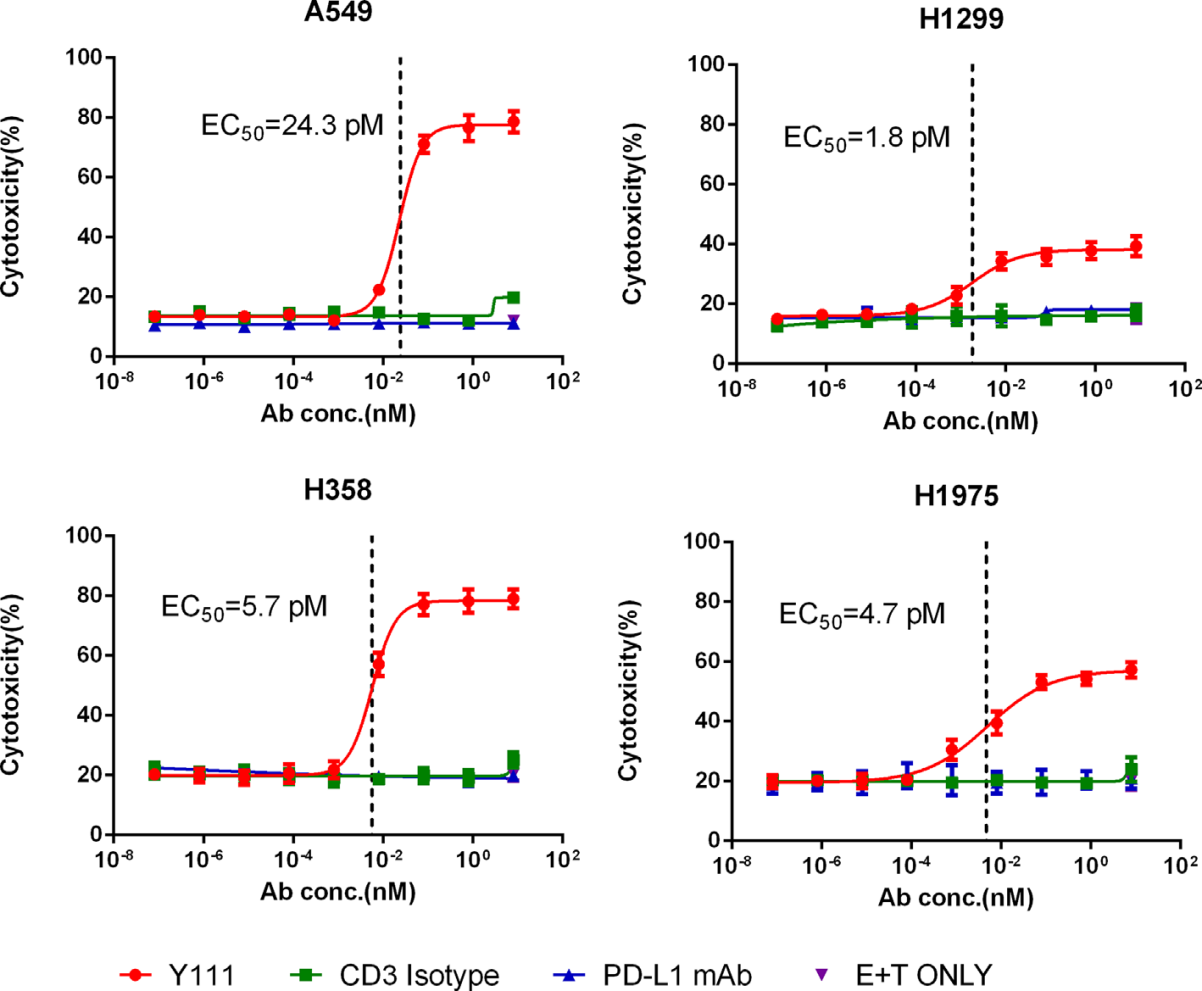
Y111 redirected Vγ2Vδ2 T cells to kill PD-L1 positive NSCLC cell lines *in vitro*. The expanded and purified Vγ2Vδ2 T cells were incubated with four NSCLC cell lines, including A549, H1299, H1975, and H358, in a 1:1 ratio with the Y111 in a range of concentrations, CD3 Isotype or PD-L1 mAb for 12 hours. Then the proportions of killed target cells were plotted against the antibody concentrations. These cell lines were PD-L1 positive shown in **Supplementary Figure 6**. The calculated EC_50_ values were shown. The data points in were represented as mean ± SEM among 9 individual subjects for the analysis.

We chose four NSCLC cell lines including A549, H1299, H358, and H1975 cells, as these four types of cancer cells express high levels of PD-L1 (**Supplementary Figure 6**). When CFSE-stained tumor cells were co-cultured with purified Vγ2Vδ2 T cells at a ratio of 1:1 and a range of serially diluted Y111 antibody for 12 hours, tumor cells were killed efficiently by Y111 in a dose-dependent manner, but not at all by CD3 Isotype or PD-L1 mAb at any tested concentration (**Figure 5**). As Y111 alone did not affect the viability of tumor cells (**Supplementary Figure 7**), it was believed that the observed high cytotoxicity was directly elicited by Y111-induced T cells. Although the anti-PD-L1 activity of Y111 may block the PD1/PD-L1 interaction and act as a checkpoint inhibitor, our data showed that the PDL1-antibody alone had little effect on the killing ability of the Vγ2Vδ2 T cells against PDL1-positive tumor cells (**Figure 5** **).** We noticed that only four pair dots might not provide meaningful correlations, but we indeed found a negative trend between the Y111-induced killing ability (EC50 values) and the PD-L1 positive percentages (**Supplementary Table 1**). Furthermore, Y111 plus the expanded Vγ2Vδ2 T cells did not attack the normal cells (such as PBMCs) from other donors (**Supplementary Figure 8**), suggesting the safety of the combination of the Y111 and the expanded Vγ2Vδ2 T cells in its potential clinical applications.

### The Secretion of IFNγ, TNFα, and Granzyme B From Vγ2Vδ2 T Cells Was Enhanced by Y111 Along With the Killing of the Tumor Cells

**Figure 6:**
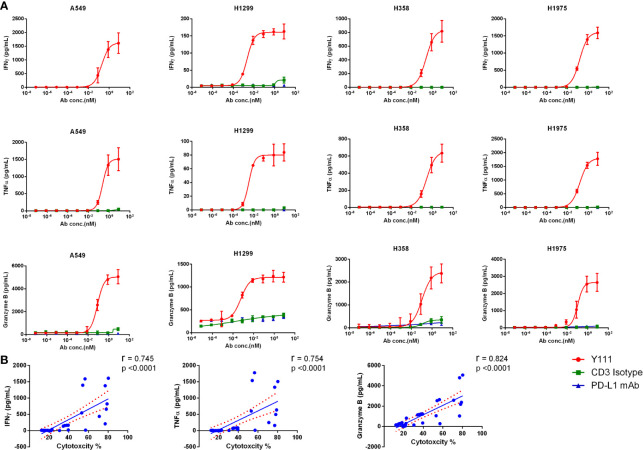
Y111 prompted the release of IFNγ, TNFα, and granzyme B from Vγ2Vδ2 T cells. **(A)** The increased cytokine secretion by Vγ2Vδ2 T cells after co-culture for 12 h with tumor cells with Y111 or control antibodies (as indicated in **Figure 4**) analyzed by the CBA method. Please note that the values were the results of raw values subtracted E+T only groups. The data points in **Figure 5** were represented as mean ± SEM among 9 healthy subjects. **(B)** The correlations of the enhanced cytotoxicity induced by Y111 with the increased production of IFNγ, TNFα, and granzyme **(B)** The spearman’s r and two-tailed p values were calculated by GraphPad prism 6. The blue line indicated the best-fit line, and the red line indicated the 95% confidence band of the best-fit line.

The killing ability of Vγ2Vδ2 T cells induced by Y111 prompted us to check the production of killing cytokines, including IFNγ and TNFα, and cytotoxic mediator granzyme B in the co-culture of the T cell and tumor cells. We found Y111, but not CD3 Isotype or PD-L1 mAb could significantly enhance the secretions of IFNγ, TNFα, and granzyme B from the expanded Vγ2Vδ2 T cells in the presence of tumor cells (**Figure 6A**). Moreover, the evaluated releases of IFNγ and TNFα, and granzyme B were consistent with the enhanced killing ability of the Vγ2Vδ2 T cells mediated by Y111, as inferred from the significant correlation coefficients between the secreted amounts of IFNγ, TNFα, and granzyme B and the cytotoxicity activities (**Figure 6B**). However, there is no obvious increase of IFNγ and TNFα in the co-cultures of the expanded and purified Vγ2Vδ2 T cells and PBMCs from other donors in the presence of Y111 (**Supplementary Figure 8**).

### Adoptive Transfer of the Purified and Expanded Vγ2Vδ2 T Cells With Y111 Displayed Potent Anti-Tumor Efficacy in NPG Mice

**Figure 7:**
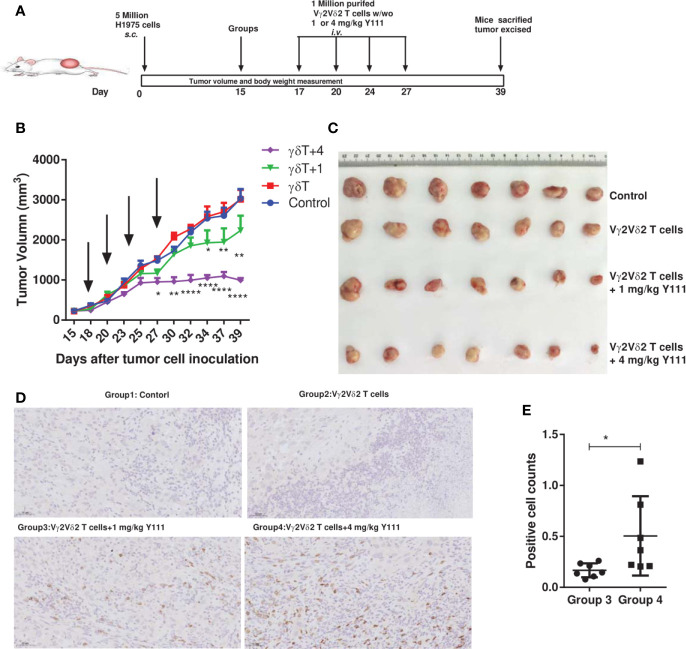
The combined usage of transfused Vγ2Vδ2 T cells with Y111 significantly inhibited tumor growth *in vivo*. **(A)** Experimental schema of protocols for establishing xenograft in NPG mice and evaluating the anti-tumor therapeutic efficacy of different treatments. Immunodeficient NPG mice were *s.c.* inoculated with H1975 NSCLC cells on Day 0. After seventeen days, mice were treated with *i.v.* transfused Vγ2Vδ2 T cells w/wo 1 or 4 mg/kg Y111. These treatments were repeated twice per week for 2 weeks. Mice treated PBS were used as control. **(B)** The pooled tumor growth curves for NPG mice in four groups. The black arrows indicated the treatment time point. Data are mean ± SEM with 7 mice per group, ****p < 0.0001, **p < 0.01, *p < 0.05 versus control group, two-way ANOVA followed by Dunnett test. **(C)** Inspection of tumor tissues excised from each group at the end of the study. **(D)** Representative IHC photomicrographs of tumors excited from mice stained with the anti-human CD3 antibody. Magnification, 20X. **(E)** Infiltrated and accumulated T-cell counts at the tumor sites for mice received purified Vγ2Vδ2 T cells with 1 or 4 mg/kg Y111. Quantitative analysis of Vγ2Vδ2 T cells was done by counting positive dots in a total of 70 fields from 14 mice. We did not find the accumulation of Vγ2Vδ2 T cells in the other two groups. Each dot represented one mouse. Data were presented as mean ± SEM, *p < 0.05, Mann Whitney U test.

To assess the therapeutic potential of transfusing Vγ2Vδ2 T cells with bispecific antibody Y111, we utilized the H1975-NPG model to check whether this combination treatment could fight against the established xenograft in mice model (**Figure 7A**). Adoptive transfer of the *ex vivo* expanded and purified Vγ2Vδ2 T cells alone had no effect on the growth of the established H1975-derived xenograft, similar to the control group (**Figures 7B, C**, **Supplementary Figure 9A**). In contrast, the supplementation of the Y111 combined with Vγ2Vδ2 T cells purified from the same donor significantly delayed the malignant progression, comparing to the control or the T cells alone groups (**Figures 7B, C** and **Supplementary Figures 9A**). These significant inhibitory effects of tumor growth started on Day 27 after tumor cell inoculation in the mice received both Vγ2Vδ2 T cells and 4 mg/kg Y111 (**Figure 7B** and **Supplementary Figure 9A**). Moreover, 4 mg/kg Y111 elicited superior suppressive effects with a greater extent of delaying tumor growth of this group than 1 mg/kg Y111 group (**Figures 7B, C** and **Supplementary Figure 9A**). The observed inhibitory effects were associated with significant increases in the infiltration and accumulation of transfused Vγ2Vδ2 T cells induced by Y111 (**Figures 7D, E**). During the study, the Y111 treatment resulted in no or little weight loss in mice (**Supplementary Figure 9B**). All of these results demonstrated that Y111 enhanced the anti-tumor efficacy of transfused Vγ2Vδ2 T cells, suggesting a potential safety and efficient therapy of the combination of the expanded Vγ2Vδ2 T cells with the Y111 bsAb.

## Discussion

Since the discovery of the Vγ2Vδ2 T cells in the late 1980s, a significant amount of knowledge has been accumulated concerning its vital roles in killing tumor cells and controlling tumor growth, raising the possibility of its potential for anti-cancer therapeutics (35–37). The currently available results of clinical trials using the transferred Vγ2Vδ2 T cells against both hematological malignancies and solid tumors were proved to be safe but ineffective (35, 38). The low efficacy results could be due to the failure of the transfused Vγ2Vδ2 T cells infiltrating into tumor sites or due to the suppression of the killing activity of the transfused Vγ2Vδ2 T cells by the tumor microenvironment (4, 39). In this study, the transfused Vγ2Vδ2 T cell was redirected into tumor sites by a novel anti-CD3 and anti-PD-L1 bsAb, Y111. This proof-of-concept study also verified the value of the bsAb-based immunotherapy to leverage the potent anti-tumor capacity of Vγ2Vδ2 T cells, and suggested that the combination of the Vγ2Vδ2 T cells and Y111 could be applied for PD-L1+ cancer therapies.

With the obligatory ability in two binding specificities simultaneously, bispecific antibodies are progressing into clinical developments for a wide variety of tumors (14, 26). In this study, we generated a PD-L1 X CD3 bsAb Y111, based on the Y-body^®^ technological platform, which was characterized as an asymmetric format for easy purification, with the modified Fc fragment to abolish Fc-mediated effector functions (25, 26). The observed MW of Y111 was larger than the theoretical MW, as a result of its N-linked glycosylation, which prompted its stability (25). Moreover, the Y111 retained a relatively weaker binding affinity to the CD3 molecule, comparing to its parent monoclonal antibody 2A5, but displayed a similar affinity to PD-L1 as that of its parental mAb. The reduced affinity for CD3 of Y111 was desired for clinical applications as several previous studies had shown that a lower affinity of the anti-CD3 moiety of a T cells-redirecting bsAb contributed to the efficient tumor infiltration of the T cells without rapid CD3-modulated plasma clearance (40–42), and to lowing the risk of cytokine release syndrome (CRS) (25, 26). Indeed, our data indicated that Y111 could prompt T cell infiltration into tumor sites *in vivo* and induced high potential cytotoxicity against tumor cells *in vitro*.

The different susceptibility of fresh CD8+ and Vδ2 T cells-, and the expanded Vγ2Vδ2 T cells-modulated the killing activities of tumor cells induced by Y111 may be attributed to the various action mechanisms of the TCR activation by these cells (43, 44). The observation in this study indicated that the cytotoxicity of the fresh Vγ2Vδ2 T cells would not be enhanced by Y111. As the adoptive transfer of the expanded Vγ2Vδ2 T cells was proved to be safe and well-tolerated in clinical evaluation, here we showed that the combination of the expanded Vγ2Vδ2 T cells and Y111 would improve the efficacy of the current therapy. Indeed, our data demonstrated that Y111 triggered the up-regulated expression of CD107a on the surfaces of Vγ2Vδ2 T cells and selectively provoked their production of IFNγ and TNFα in the presence of PD-L1 expressing tumor cells. Moreover, the observed killing of PD-L1 expressing NSCLC cell lines was not affected by gene variations in these tumor cell lines, including the mutations of KRAS (A549 and H358 cells) or EGFR (H1975 cells), and the loss of P53 activities (H358 and H1299 cells). This gene variation-ignored killing mechanism of our approach further proved the potential anti-tumor nature of the Vγ2Vδ2 T cells (35, 36, 45). Yet, the Y111-induced cytotoxicity of the Vγ2Vδ2 T cells was dependent on the cross-linkage of the T cells and PD-L1-positive cells (**Figure 5**). However, the combination of the Y111 plus the Vγ2Vδ2 T cells did not lyse PBMCs from the unrelated healthy donors (**Supplementary Figure 8**); no significant change of the body weights in the Y111 + Vγ2Vδ2 T cells treated mice was observed (**Supplementary Figure 9B**). These results suggested that the safety of the combination approach reminded as that of the adoptive transferred Vγ2Vδ2 T cells therapy (35, 38).

While expanding a large scale of autologous Vγ2Vδ2 T cells from a cancer patient *ex vivo* still represents a critical clinical challenge (37), we explored the antitumor activity of a modified protocol by transferring a small amount of Vγ2Vδ2 T cells together with Y111 into NPG mice bearing tumor cell line derived xenograft. The approach seems particularly promising given the potential of controlling the growth of established tumors in mice model, while the therapy of the transfused Vγ2Vδ2 T cells alone was not effective. This better efficacy *in vivo* result was consistent with the increased cytotoxicity of this treatment *in vitro*.

It is not feasible to directly using syngeneic mouse tumor models to evaluate Vγ2Vδ2 T cell-based anti-cancer therapy since the Vγ2Vδ2 T cell subset exists only in human and non‐human primates, but not in rodents (46). Due to the limitation of immunodeficiency of NPG mice used in this study, we could not probe whether our strategy could modulate suppressive tumor microenvironment. Previous study showed that Treg cell, which has a strong immunosuppressive function in tumor microenvironment, could regulate phosphoantigen-induced proliferation of Vγ2Vδ2 T cell *ex vivo*, but did not suppress the cytokine production or cytotoxic effector functions of Vγ2Vδ2 T cell (47).However, phosphoantigen+IL2-expanded Vγ2Vδ2 T cells could antagonize the expansion and functions of CD4+CD25+ regulatory T cells both *in vivo* and *in vitro* (48), and even overcome TGFβ immunosuppressive functions (49). Moreover, the clinical trials did not offer evidence of Treg-exerting immunosuppression to Vγ2Vδ2 T cells, as the repeated administration of IL2 was regarded as a standard operation (50). Thus, based on these previous reports, we believed that Tregs did not impair the killing function of the expanded Vγ2Vδ2 T cells in the presence of Y111. Recently, a series of studies have probed the cross-talk of the tumor resistance mechanisms and Vγ2Vδ2 T cells, and concluded that a combination therapy of adoptively transferred Vγ2Vδ2 T cells and bispecific T cell engagers is a possible future directions to overcome the immunosuppressive tumor microenvironment [reviewed in 5]. Consistent with this concept, we found Y111 could increase the trafficking of the transferred Vγ2Vδ2 T cells into the tumor site (**Figure 6D**) even for 12 days after the last cell transfer. Comparing to other studies using bispecific antibodies or anti-TIM3 monoclonal antibodies with the Vγ2Vδ2 T cells (7–13), our combination approach was demonstrated to be effective and safe without the additional administrations of IL2 or aminobisphoshponates or pyrophosphates for sensitizing the tumor cells.

In conclusion, this study demonstrated that bispecific antibody Y111, targeting the CD3 on Vγ2Vδ2 T cells and the PD-L1 on the tumor cells, could harness the anti-tumor potential of the Vγ2Vδ2 T cells to kill the cancer cells *in vitro* and inhibit the growth of the established xenograft tumors *in vivo*. The study provides new evidence to support the hypothesis that a CD3-targeting bispecific antibody has the potential to enhance the Vγ2Vδ2 T cells-based anti-tumor efficacy. The combination immunotherapy of the Y111 and the expanded Vγ2Vδ2 T cells is worth for further clinical evaluation for its benefit to cancer patients.

## Data Availability Statement

The raw data supporting the conclusions of this article will be made available by the authors, without undue reservation.

## Ethics Statement

The studies involving human participants were reviewed and approved by Institutional Animal Care and Use Committee at Huazhong University of Science and Technology (Wuhan, China). The patients/participants provided their written informed consent to participate in this study. The animal study was reviewed and approved by Institutional Animal Care and Use Committee at Huazhong University of Science and Technology (Wuhan, China).

## Author Contributions

RY, LG, JY, and PZ designed the project. JZ, YY, LF, and LZ supervised the project. RY, SS, ZF, YX, CG, XW, FL, ZW, LY, and FYL performed the experiments. RY, LG, JY, and PZ analyzed the data and jointly wrote the manuscript. All authors contributed to the article and approved the submitted version.

## Funding

This work was supported by the National Natural Science Foundation of China (Grant No. 81901607).

## Conflict of Interest

L.G declares no financial conflicts of interest. Others in the authorship are employees of Wuhan YZY Biopharma Co., Ltd.

The remaining author declares that the research was conducted in the absence of any commercial or financial relationships that could be construed as a potential conflict of interest.

## Supplementary Material

Supplementary Figure 1 The schematic diagrams of antibodies used in this study and the MOA of Y111. **(A)** Schematic diagrams of bispecific antibody Y111, PD-L1 mAb, and CD3 Isotype. Y111, a bispecific antibody targeting both PD-L1 and CD3; PD-L1 mAb, the parental monoclonal antibody targeting PD-L1; CD3 Isotype, a control bispecific antibody targeting CD3 and fluorescein (Clone 4420). **(B)** The proposed model for the mechanism of action (MOA) in this study. Y111 bridges the PD-L1 positive tumor cells to the Vγ2Vδ2 T cells to form the immune synapse, resulting in the release of cytolytic granzyme B, IFNγ, and TNFα.

Supplementary Figure 2 The quality of expanded Vγ2Vδ2 T cells for *in vitro* and *in vivo* assays. **(A, B)** Kinetics of population and absolute numbers of Vγ2Vδ2 T cells during the expansion (n=9). **(C)** The expression levels of PD-L1 on Vγ2Vδ2 T cells and dead cells (PI-positive) among Vγ2Vδ2 T cells at day 14 (n=9). **(D)** Representative flow cytometry plots showed the population of Vγ2Vδ2 T cells at day 0-, day 14-PBMC cultures. Then, Vγ2Vδ2 T cells were negatively isolated from the day 14-cultures. The purity of enriched Vγ2Vδ2 T cells was assessed by flow cytometry. **(E)** The expression levels of the co-stimulatory molecule CD86, the activation associated marker CD69, and antigen-presenting molecule HLA-DR on Vγ2Vδ2 T cells at day 0 (red lines) and day 14 (blue lines). The black line represents isotype controls. These enriched cells were used for either binding or killing and functional assay *in vitro* or assessing anti-tumor activity *in vivo*.

Supplementary Figure 3 Y111 bridged the tumor cell and the Vγ2Vδ2 T cells in a dose-dependent fashion. CFSE-stained H1975 cells were co-cultured with PKH26-labeled Vγ2Vδ2 T cells in the presence of Y111 or CD3 Isotype for 30 mins. Co-binding% was indicated as the percentages of the CFSE and PKH26 double-positive cells (Q2) of the total cells. Representative co-binding dot plots were shown in **(A)**, and a nonlinear regression depicting the dose-dependent association of Y111 was shown in **(B)**.

Supplementary Figure 4 Representative contour plots showing the production of various cytokines by Vγ2Vδ2 T cells activated by 1 μg/mL of Y111 or CD3 Isotype with or without tumor cells. The gating strategies of positive cytokines were based on the biology control with the expanded Vγ2Vδ2 T cells treated by CD3 Isotype (the third column).

Supplementary Figure 5 Multi-functional phenotypes of Vγ2Vδ2 T cells activated by Y111 or CD3 Isotype in the absence/presence of H1975 cells. After gating cytokine positive population (**Supplementary Figure 2**), the boolean analysis was utilized to check the percentages of multi-functional effector subsets (three-, or two-positive cytokines producing cells) of Vγ2Vδ2 T cells. Then the percentages of these multi-functional effector subsets of Vγ2Vδ2 T cells from four groups were plotted along with serially diluted antibodies. The shown data were the means of nine individuals of healthy subjects.

Supplementary Figure 6 The expressions of PD-L1 on four cell lines. The surface expression of PD-L1 (clone: 29E.2A3, Biolegend, San Diego, USA) on four tumor cell lines (H358, H1975, H1299, and A549) was depicted as histograms from one representative assay. The PD-L1 positive percentages of these four cell lines were 23.9%, 34.0%, 91.9%, and 93.0%, respectively. The PD-L1 positive percentages displayed a positive correlation with the Y111-induced killing ability (see **Figure 5**).

Supplementary Figure 7 Antibodies alone did not influence the viability of tumor cells. Bar graph showing Y111, CD3 Isotype, and PD-L1 mAb at 10 μg/mL exerted no effect on the growth of tumor cells. Data were from 3 independent experiments, and compared to the blank control there was no significant difference of these groups analyzed by one-way ANOVA.

Supplementary Figure 8 Y111 induced minimal lysis of PBMC mediated by the expanded and purified Vγ2Vδ2 T cells. **(A)** Vγ2Vδ2 T cells were co-cultured with CFSE-labeled unrelated PBMC in the presence of antibodies at indicated concentrations for 12 hours. Then, the killed PBMC was determined by PI staining. **(B)** Y111 failed to induce significant release of cytokines at any tested concentrations. This experiment was performed three times involving the PBMCs from four unrelated subjects as target cells.

Supplementary Figure 9 The combination usage of the transfused Vγ2Vδ2 T cells together with Y111 inhibited significantly the tumor growth *in vivo*. **(A)** Curves of tumor burden for the individual mouse from the control group against the Vγ2Vδ2 T cells only group, the Vγ2Vδ2 T cells plus 1 mg/kg of Y111 group, or the Vγ2Vδ2 T cells plus 4 mg/kg of Y111 group. **(B)** Curves of body weight changes for each group. The black arrows indicated the treatment time point. Data are the mean ± SEM with 7 mice per group.

Supplementary Table 1 The negative trend between Y111-induced killing ability (EC_50_) and PD-L1 positive percentages of tumor cell lines. a. The Pearson’s r and p-value was calculated as rMFI and EC_50_, PD-L1 positive percentages and EC_50_.

## Abbreviations

ADCC, antibody-dependent cell-mediated cytotoxicity; ATCC, American Type Culture Collection; BsAb, bispecific antibody; CBA, cytometric bead array; CFSE, carboxyfluorescein succinimidyl ester; EGFR, epidermal growth factor receptor; Fab, antigen-binding fragment; ICS, intracellular cytokine staining; IHC, Immunohistochemistry; IFNγ, interferon Gamma; NPG, NOD.Cg-Prkdc^scid^ IL2rg^tm1Vst^/Vst; NSCLC, non-small cell lung cancer; PBMCs, peripheral blood mononuclear cells; PD1, programmed cell death protein 1; PD-L1, programmed death-ligand 1; PI, propidium iodide; TNFα, tumor necrosis factor alpha; SDS-PAGE, sodium dodecyl sulfate-polyacrylamide gel electrophoresis; scFv, single-chain variable fragment.
