# Supplementary figures and images for: Bispecific Antibody PD-L1 x CD3 Boosts the Anti-Tumor Potency of the Expanded Vγ2Vδ2 T Cells
Source: Front Immunol. 2021 May 10;12:654080. doi: 10.3389/fimmu.2021.654080 (PMC8141752; doi:10.3389/fimmu.2021.654080)

**A**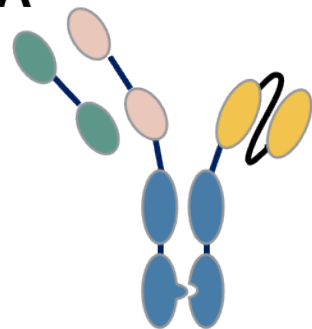

Y111  
PD-L1 X CD3

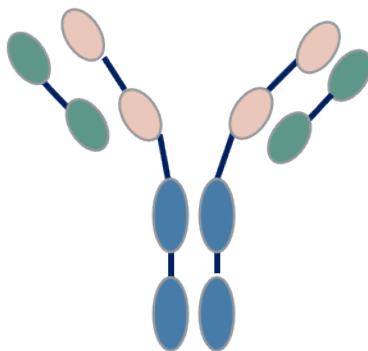

PD-L1 mAb

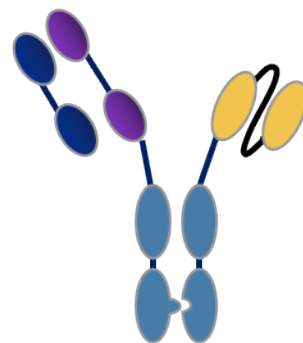

CD3 Isotype  
4420 X CD3

**B**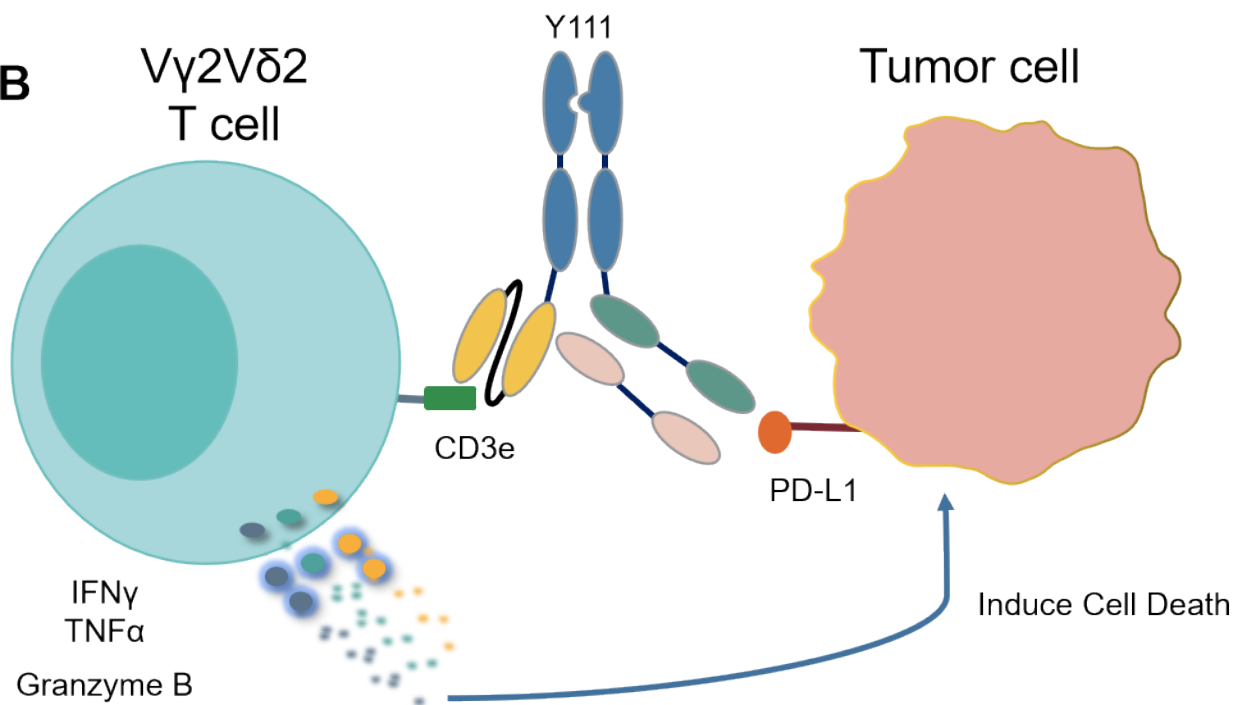

Supplement: Supplementary Figure 1 — The schematic diagrams of antibodies used in this study and the MOA of Y111. (A) Schematic diagrams of bispecific antibody Y111, PD-L1 mAb, and CD3 Isotype. Y111, a bispecific antibody targeting both PD-L1 and CD3; PD-L1 mAb, the parental monoclonal antibody targeting PD-L1; CD3 Isotype, a control bispecific antibody targeting CD3 and fluorescein (Clone 4420). (B) The proposed model for the mechanism of action (MOA) in this study. Y111 bridges the PD-L1 positive tumor cells to the Vγ2Vδ2 T cells to form the immune synapse, resulting in the release of cytolytic granzyme B, IFNγ, and TNFα. [file DataSheet_1.pdf]

**A**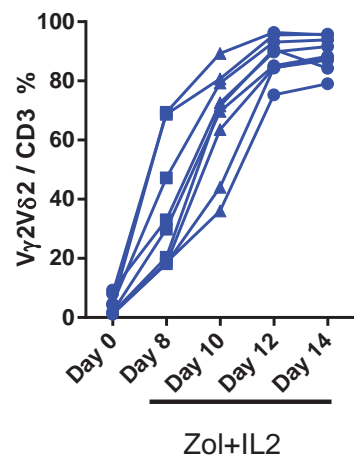**B**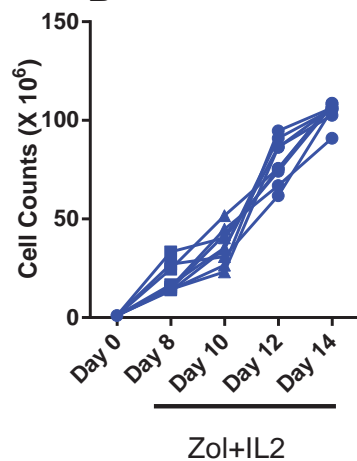**C**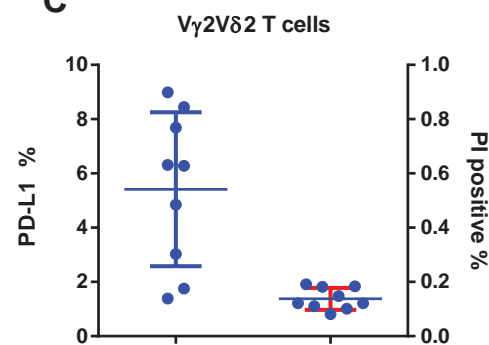**D**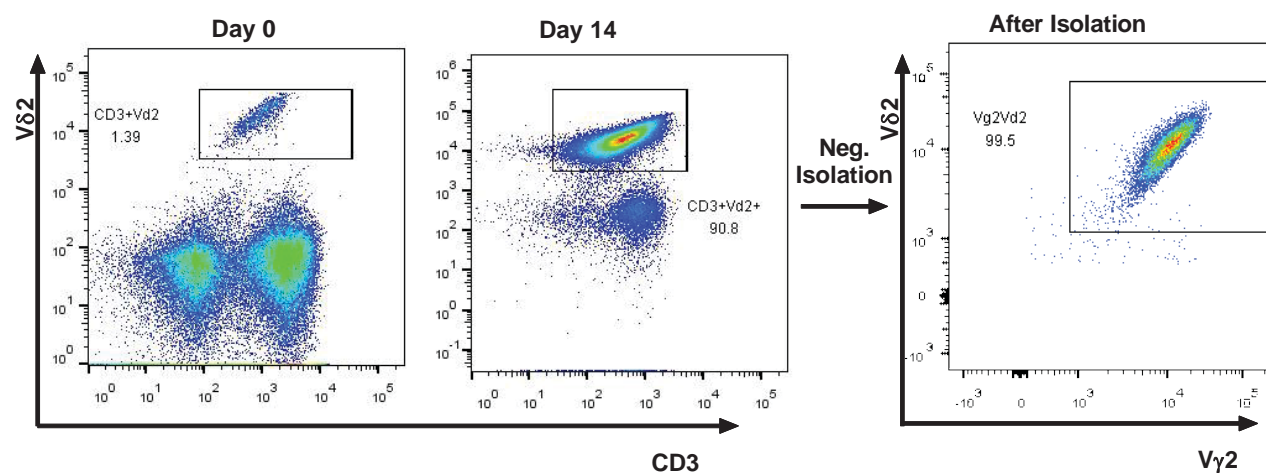**E**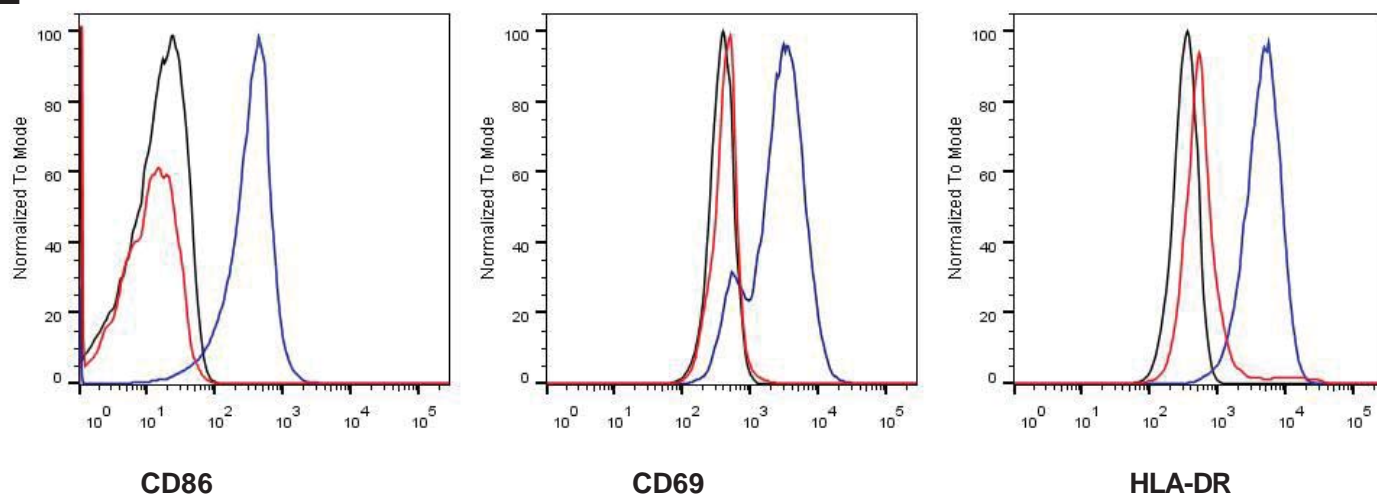

Supplement: Supplementary Figure 2 — The quality of expanded Vγ2Vδ2 T cells for in vitro and in vivo assays. (A, B) Kinetics of population and absolute numbers of Vγ2Vδ2 T cells during the expansion (n=9). (C) The expression levels of PD-L1 on Vγ2Vδ2 T cells and dead cells (PI-positive) among Vγ2Vδ2 T cells at day 14 (n=9). (D) Representative flow cytometry plots showed the population of Vγ2Vδ2 T cells at day 0-, day 14-PBMC cultures. Then, Vγ2Vδ2 T cells were negatively isolated from the day 14-cultures. The purity of enriched Vγ2Vδ2 T cells was assessed by flow cytometry. (E) The expression levels of the co-stimulatory molecule CD86, the activation associated marker CD69, and antigen-presenting molecule HLA-DR on Vγ2Vδ2 T cells at day 0 (red lines) and day 14 (blue lines). The black line represents isotype controls. These enriched cells were used for either binding or killing and functional assay in vitro or assessing anti-tumor activity in vivo. [file DataSheet_2.pdf]

**A**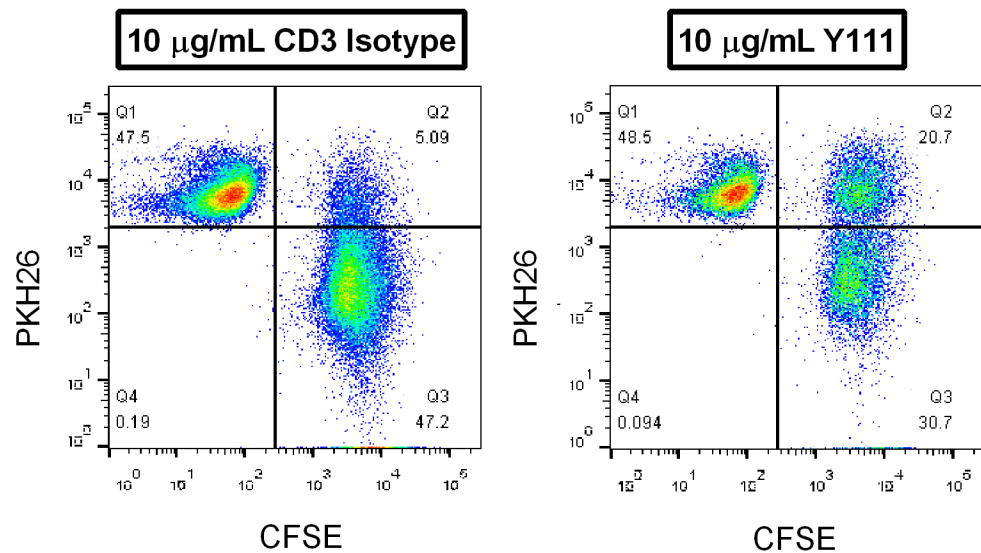**B**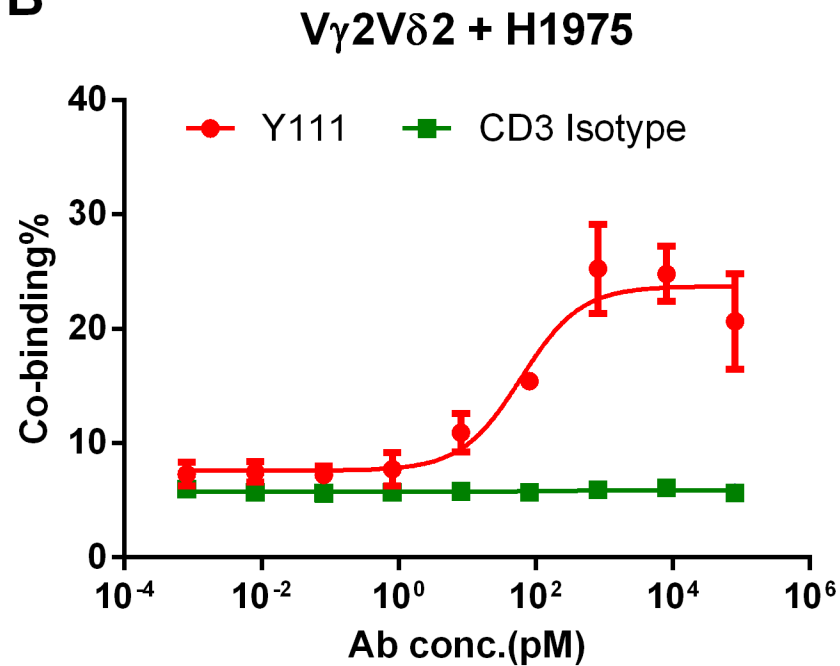

Sup.Fig.3

Supplement: Supplementary Figure 3 — Y111 bridged the tumor cell and the Vγ2Vδ2 T cells in a dose-dependent fashion. CFSE-stained H1975 cells were co-cultured with PKH26-labeled Vγ2Vδ2 T cells in the presence of Y111 or CD3 Isotype for 30 mins. Co-binding% was indicated as the percentages of the CFSE and PKH26 double-positive cells (Q2) of the total cells. Representative co-binding dot plots were shown in (A), and a nonlinear regression depicting the dose-dependent association of Y111 was shown in (B). [file DataSheet_3.pdf]

With H1975 cells

Without H1975 cells

CD3 Isotype

Y111

● ● ● ● ●

CD3 Isotype

Y111

● ● ● ● ●

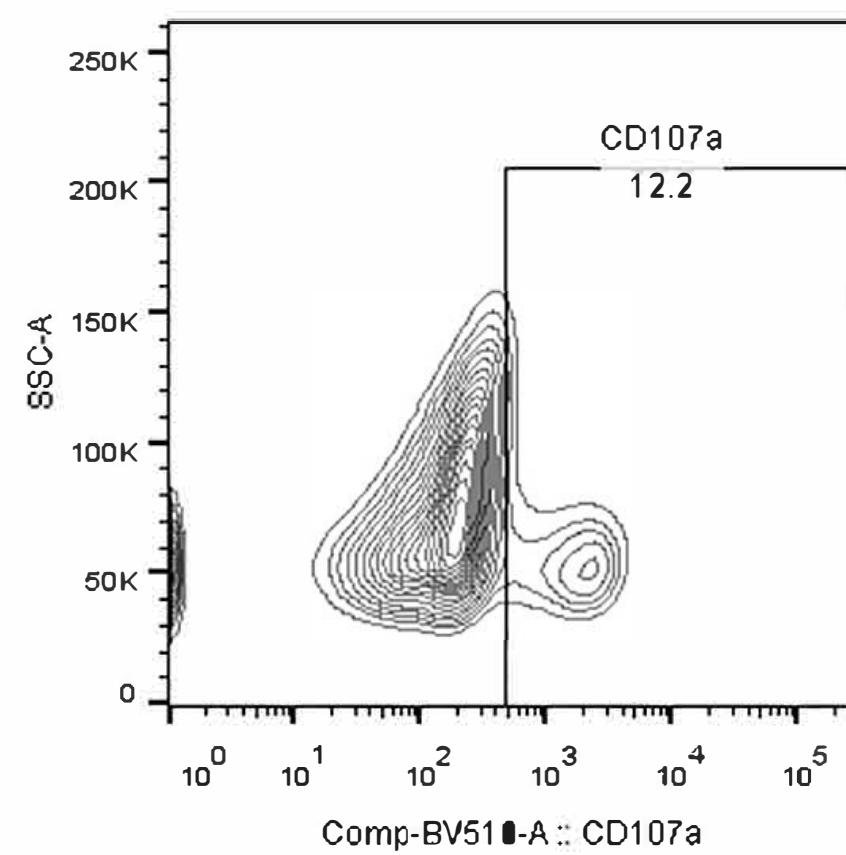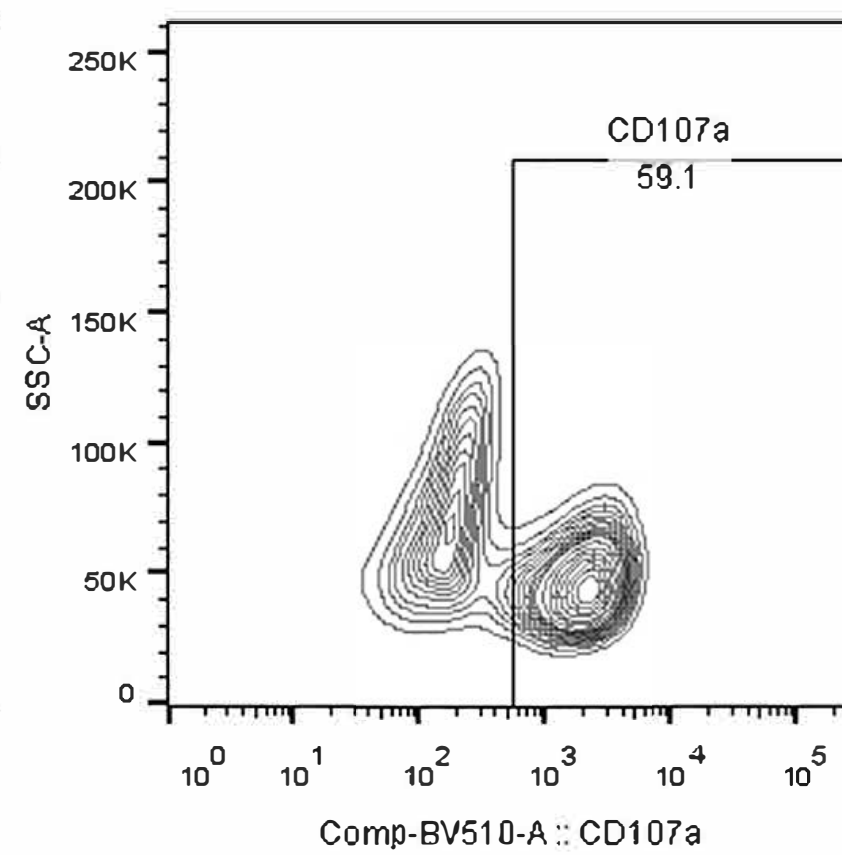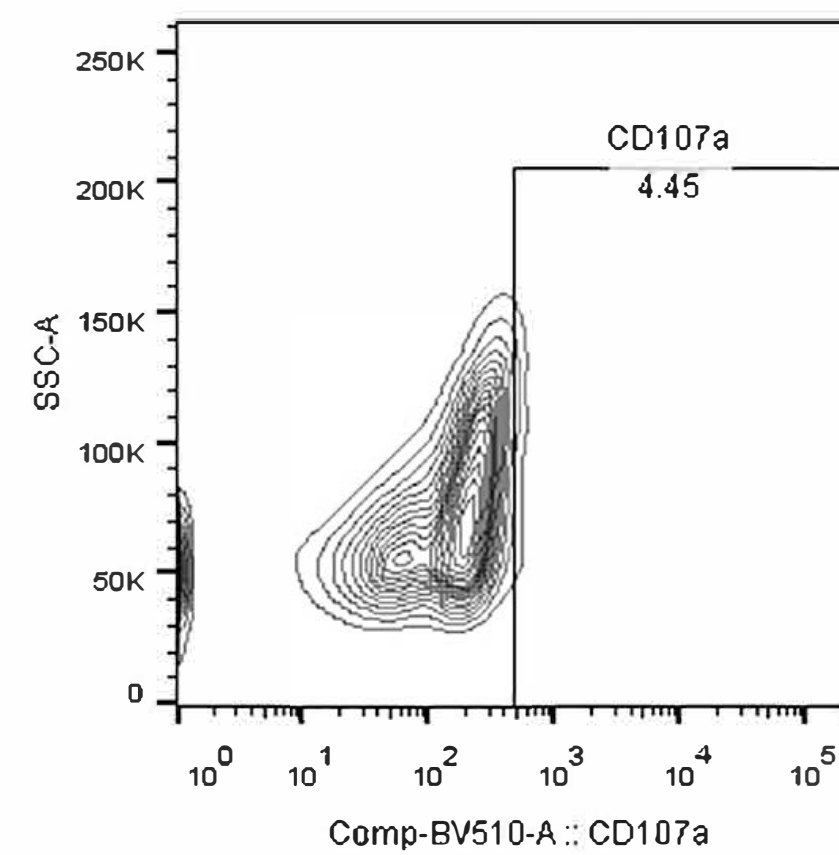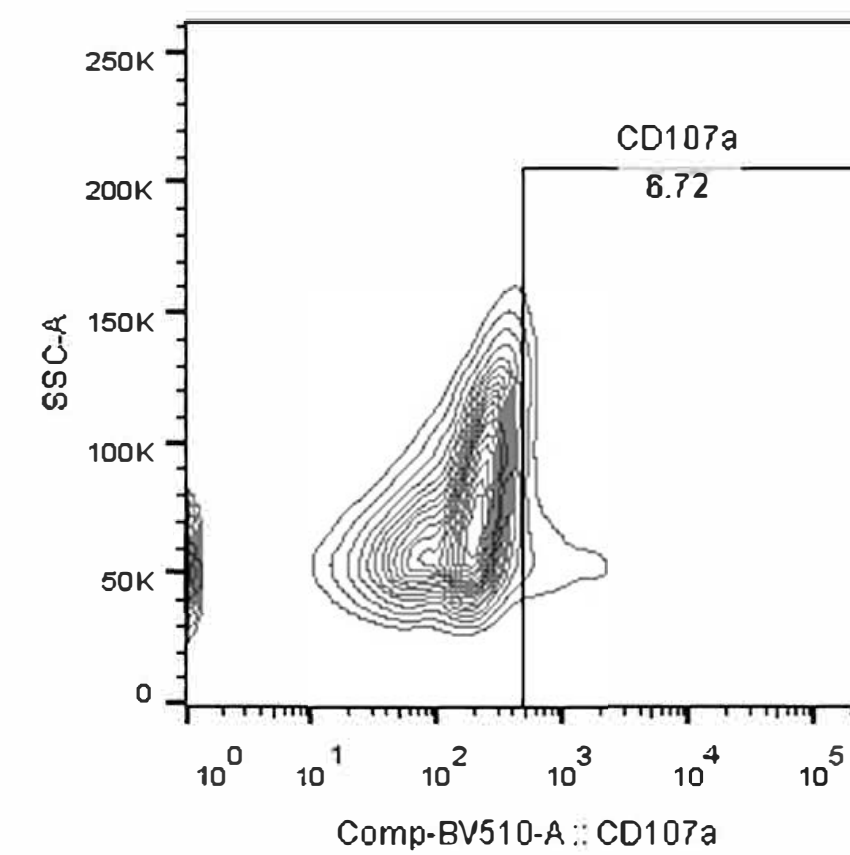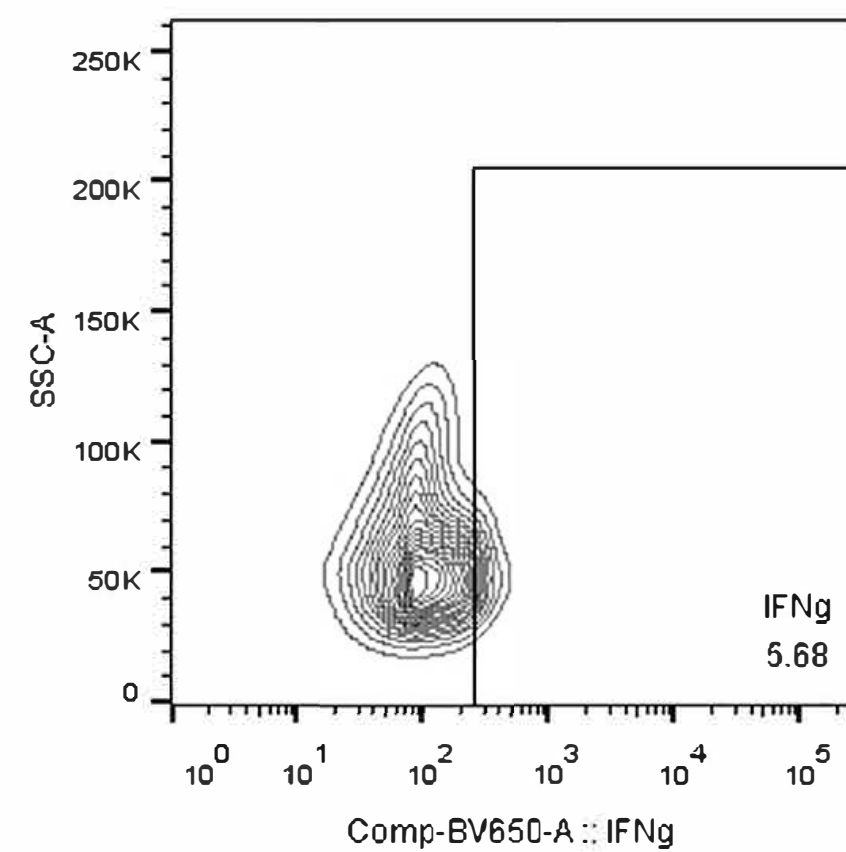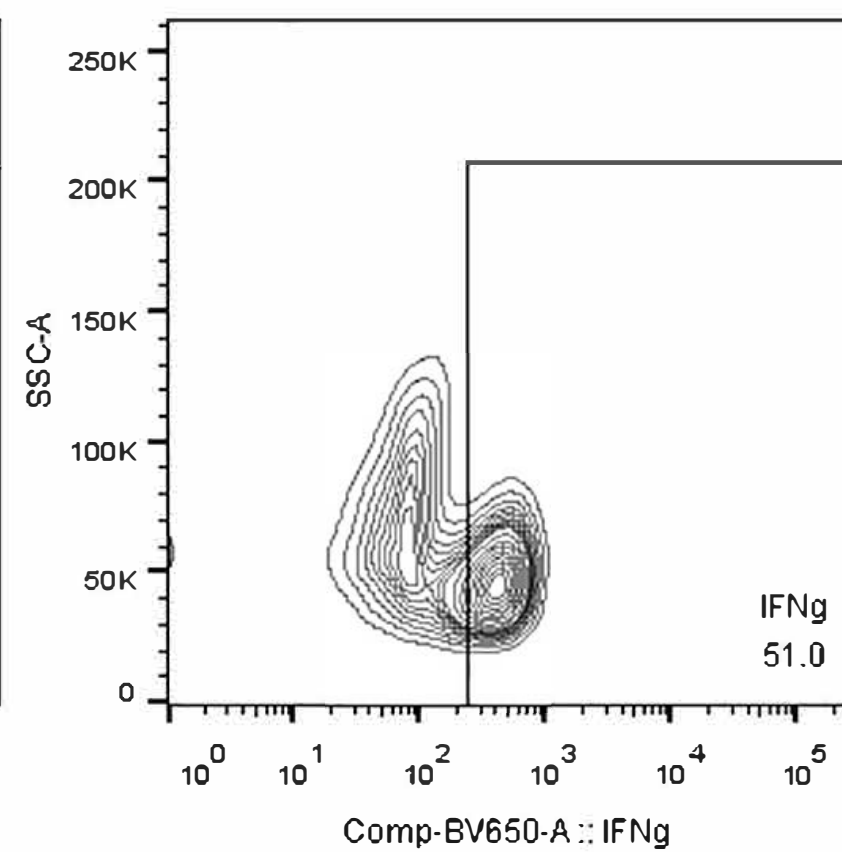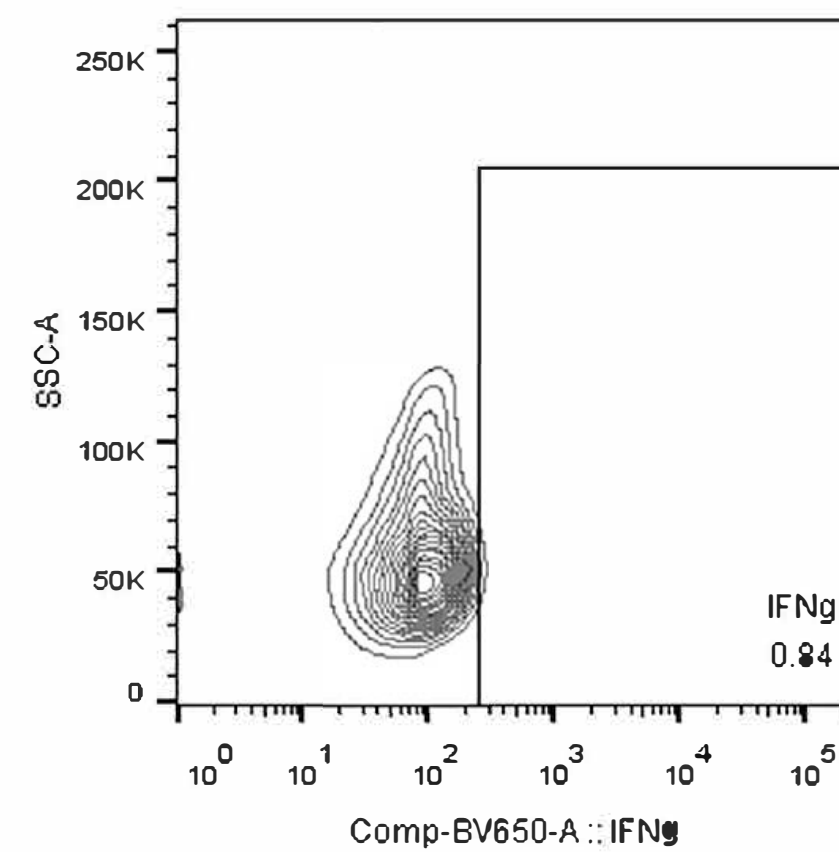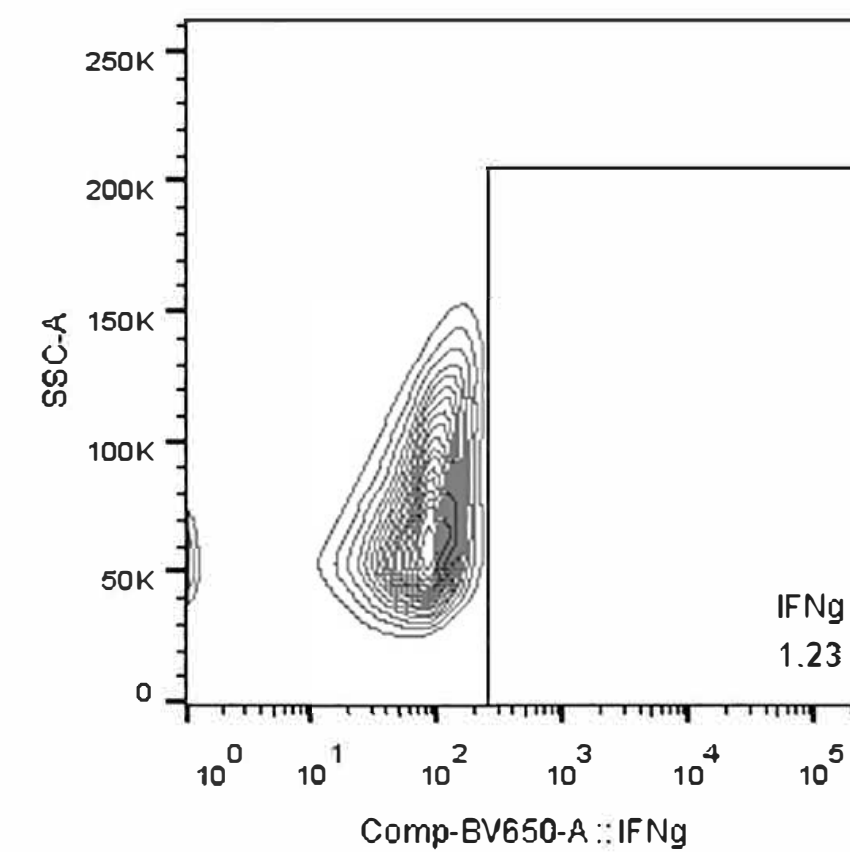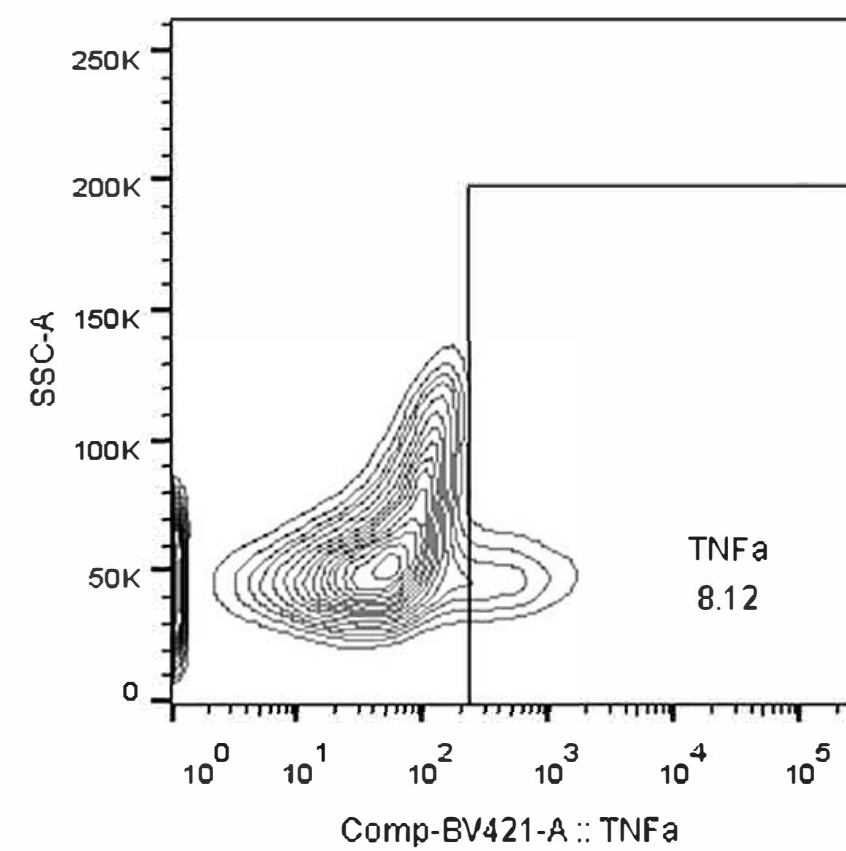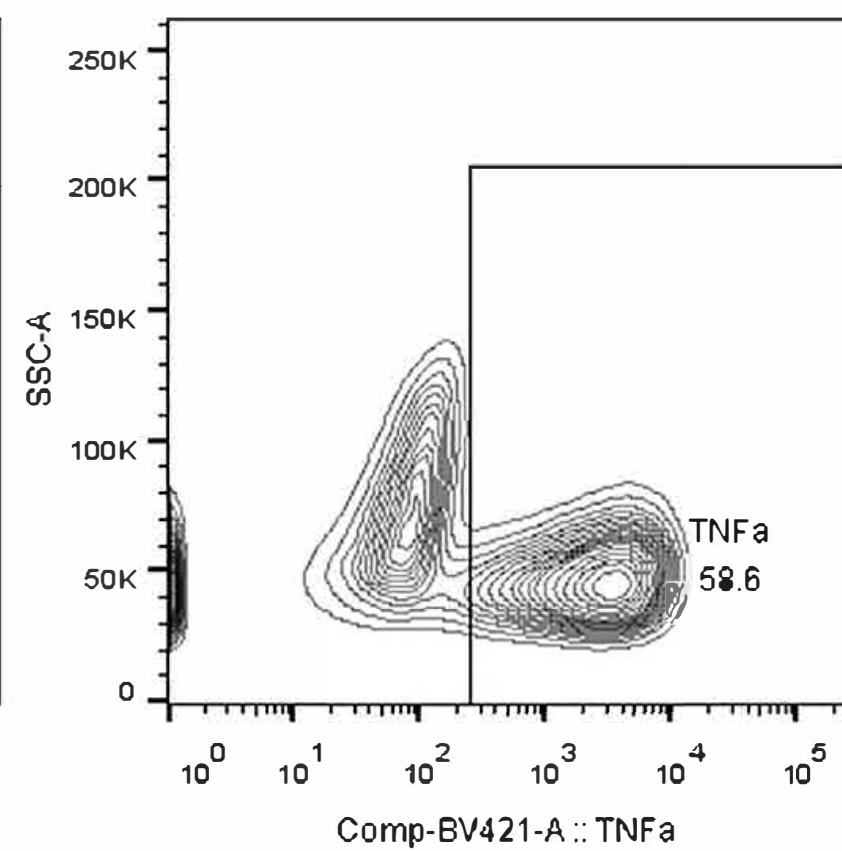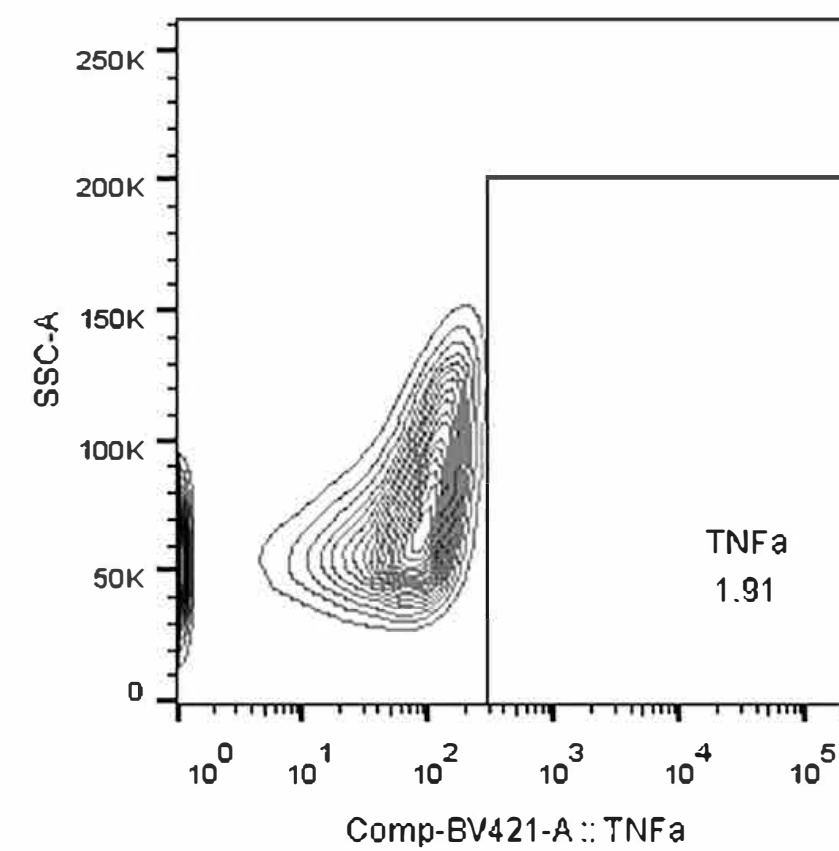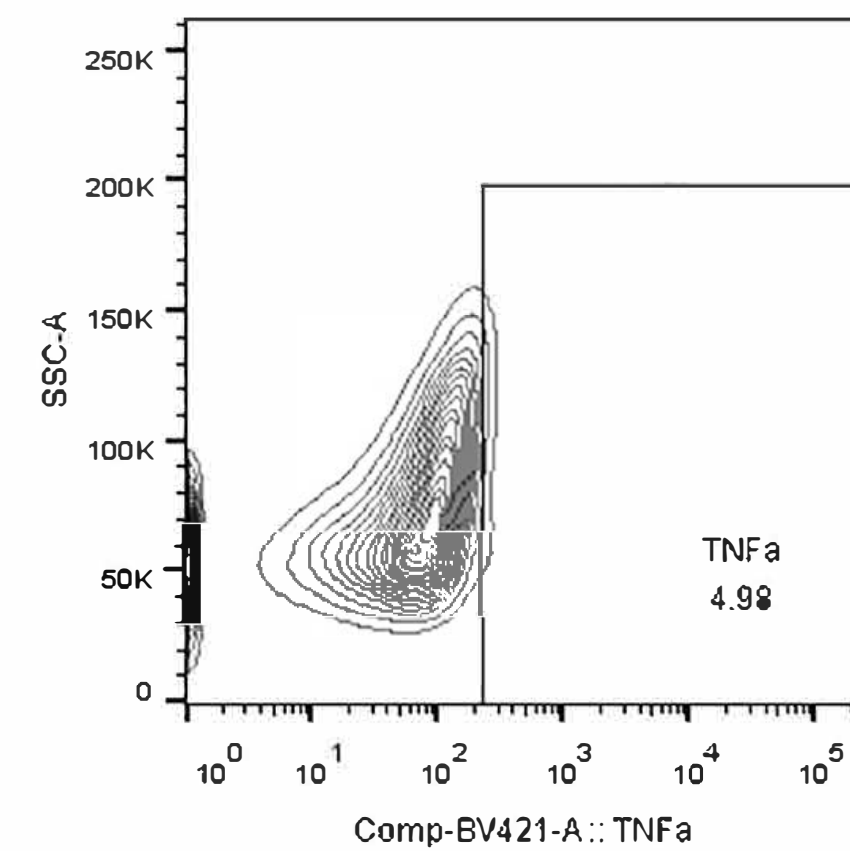

Supplement: Supplementary Figure 4 — Representative contour plots showing the production of various cytokines by Vγ2Vδ2 T cells activated by 1 μg/mL of Y111 or CD3 Isotype with or without tumor cells. The gating strategies of positive cytokines were based on the biology control with the expanded Vγ2Vδ2 T cells treated by CD3 Isotype (the third column). [file DataSheet_4.pdf]

# Mult-functional $V\gamma 2V\delta$ T cells

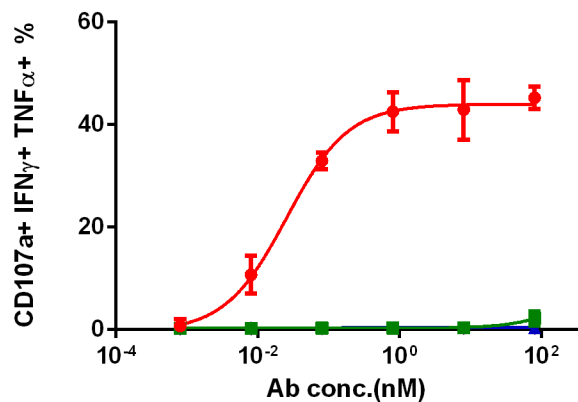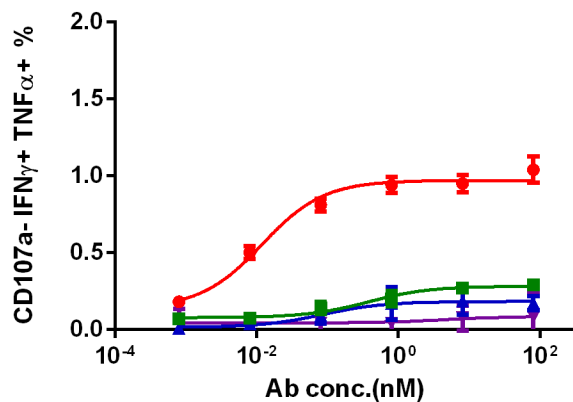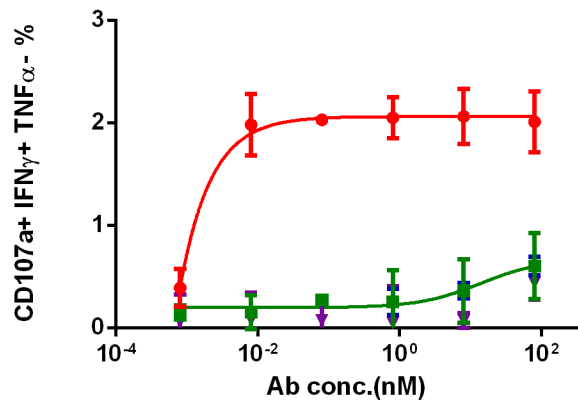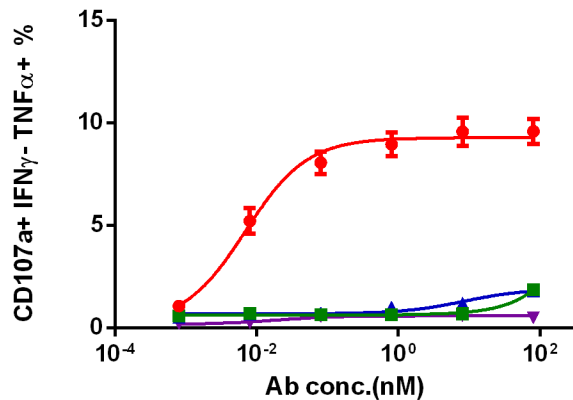

● Y111  
■ CD3 Isotype

▲ Y111  
▼ CD3 Isotype

]+H1975 cells

Supplement: Supplementary Figure 5 — Multi-functional phenotypes of Vγ2Vδ2 T cells activated by Y111 or CD3 Isotype in the absence/presence of H1975 cells. After gating cytokine positive population ( Supplementary Figure 2 ), the boolean analysis was utilized to check the percentages of multi-functional effector subsets (three-, or two-positive cytokines producing cells) of Vγ2Vδ2 T cells. Then the percentages of these multi-functional effector subsets of Vγ2Vδ2 T cells from four groups were plotted along with serially diluted antibodies. The shown data were the means of nine individuals of healthy subjects. [file DataSheet_5.pdf]

Sup.Fig.6

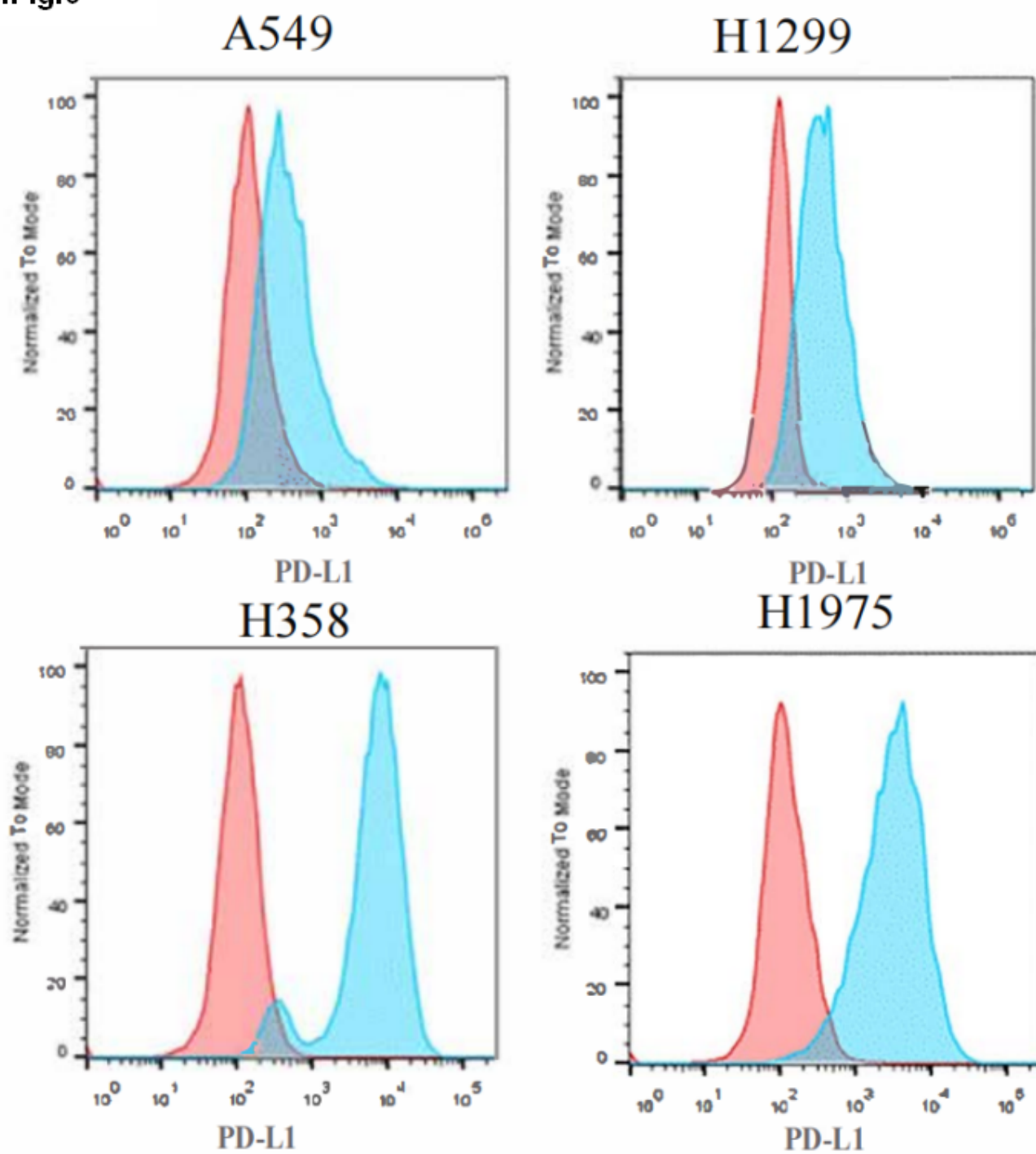

Supplement: Supplementary Figure 6 — The expressions of PD-L1 on four cell lines. The surface expression of PD-L1 (clone: 29E.2A3, Biolegend, San Diego, USA) on four tumor cell lines (H358, H1975, H1299, and A549) was depicted as histograms from one representative assay. The PD-L1 positive percentages of these four cell lines were 23.9%, 34.0%, 91.9%, and 93.0%, respectively. The PD-L1 positive percentages displayed a positive correlation with the Y111-induced killing ability (see Figure 5 ). [file DataSheet_6.pdf]

**H1975**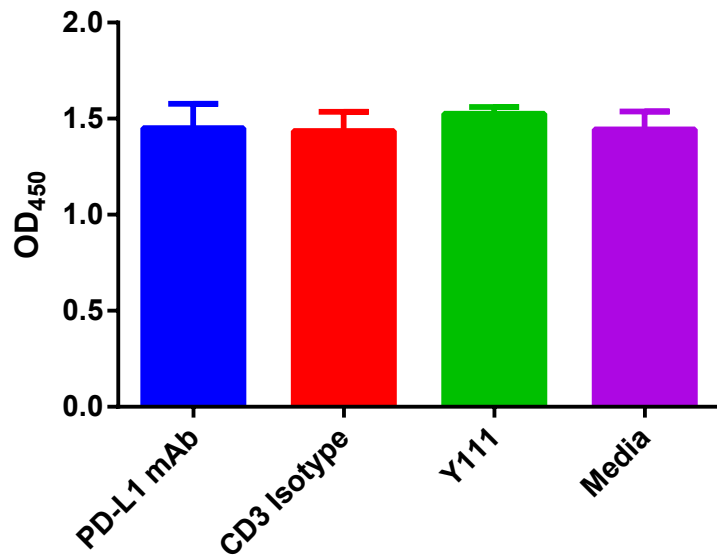**H358**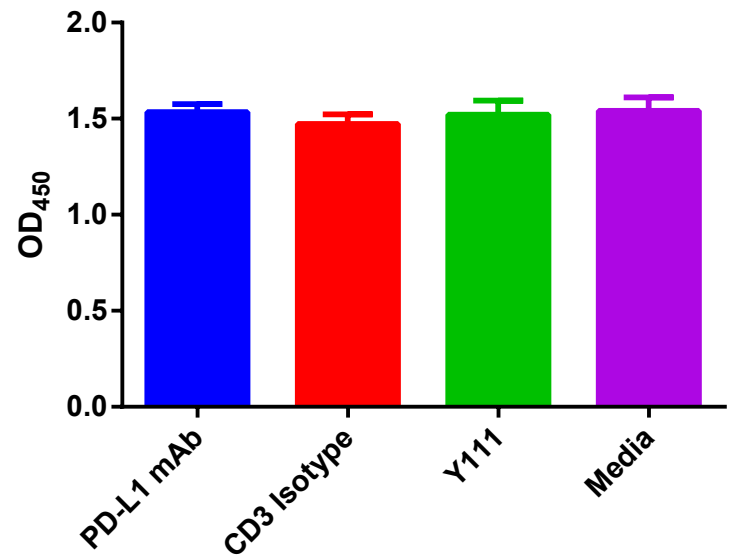**A549**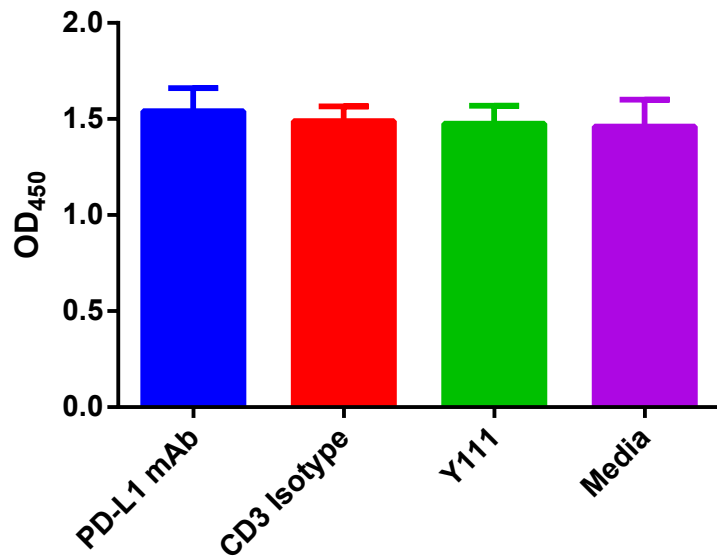**H1299**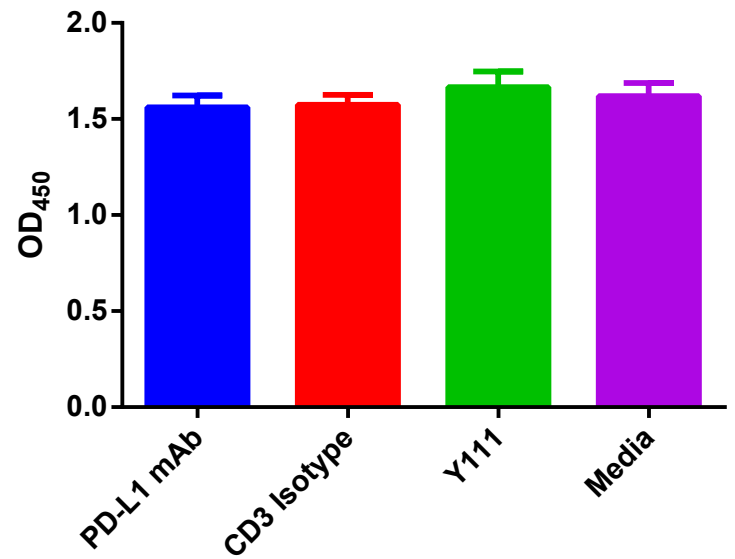

Supplement: Supplementary Figure 7 — Antibodies alone did not influence the viability of tumor cells. Bar graph showing Y111, CD3 Isotype, and PD-L1 mAb at 10 μg/mL exerted no effect on the growth of tumor cells. Data were from 3 independent experiments, and compared to the blank control there was no significant difference of these groups analyzed by one-way ANOVA. [file DataSheet_7.pdf]

**A**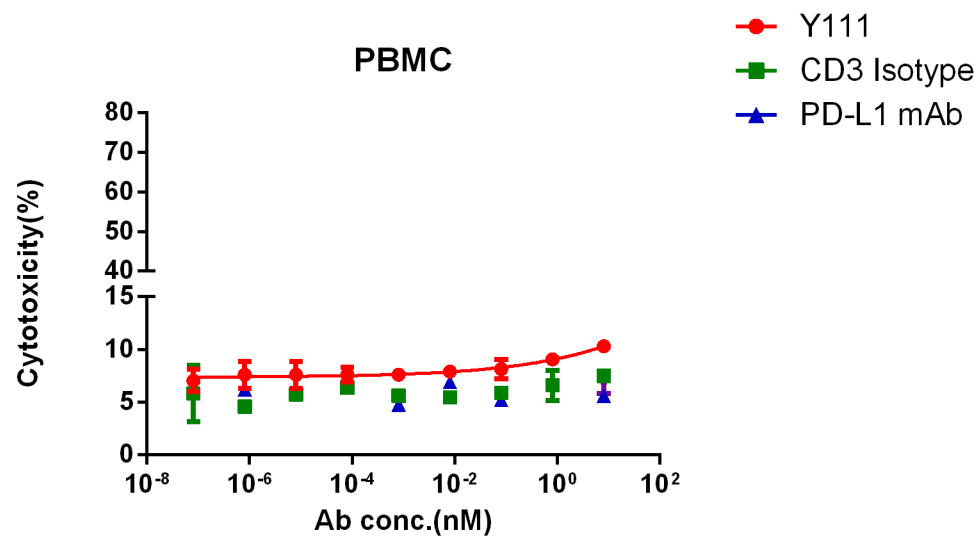**B**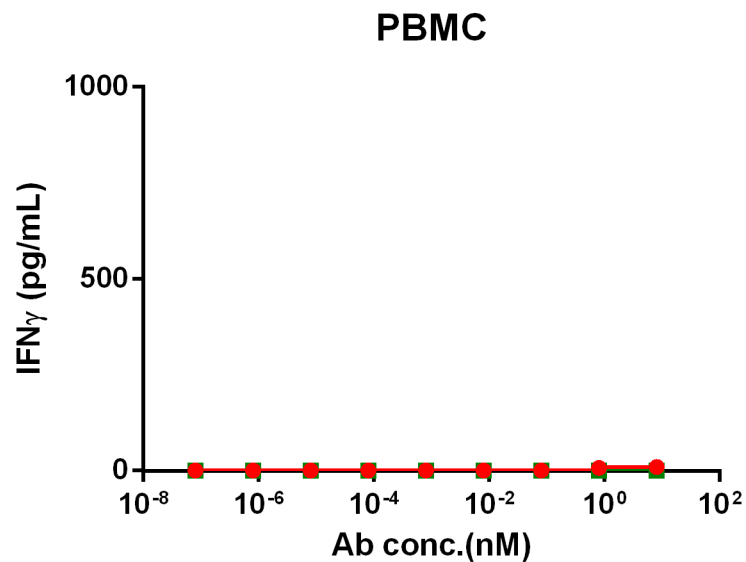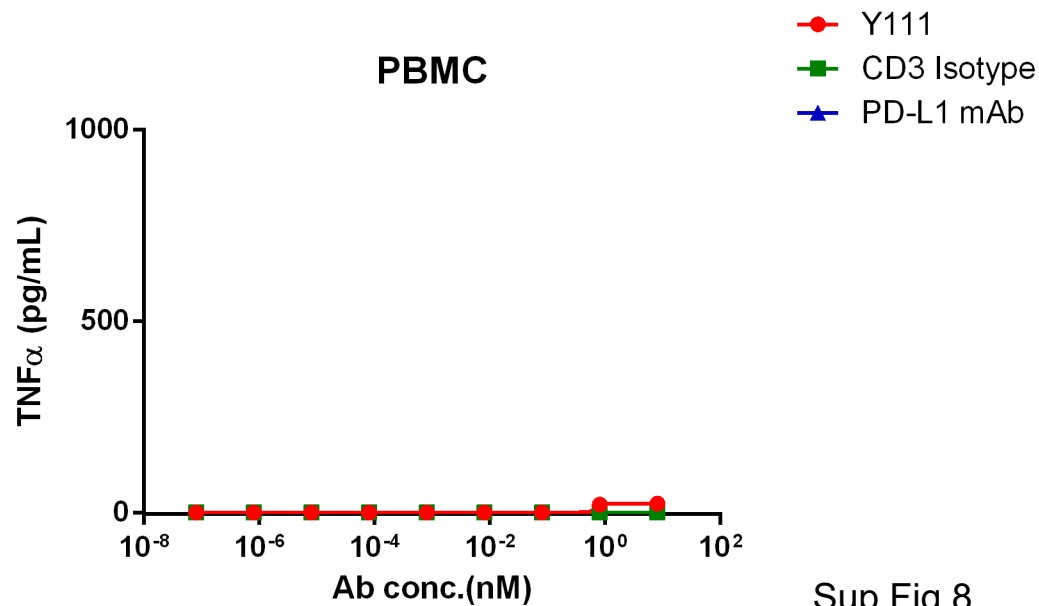

Supplement: Supplementary Figure 8 — Y111 induced minimal lysis of PBMC mediated by the expanded and purified Vγ2Vδ2 T cells. (A) Vγ2Vδ2 T cells were co-cultured with CFSE-labeled unrelated PBMC in the presence of antibodies at indicated concentrations for 12 hours. Then, the killed PBMC was determined by PI staining. (B) Y111 failed to induce significant release of cytokines at any tested concentrations. This experiment was performed three times involving the PBMCs from four unrelated subjects as target cells. [file DataSheet_8.pdf]

**A**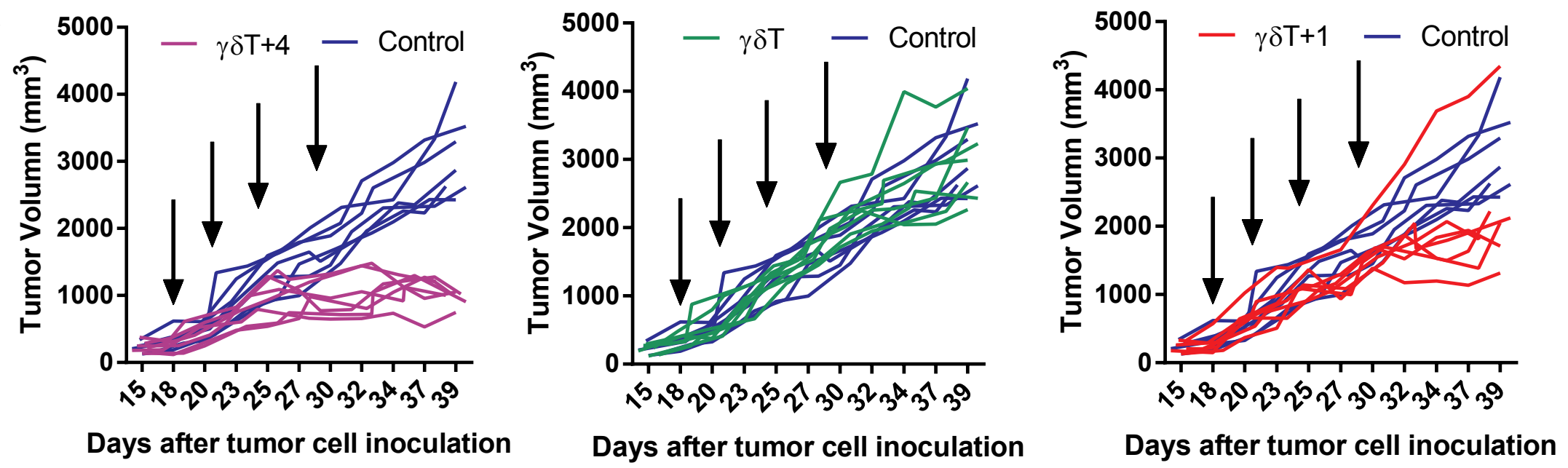**B**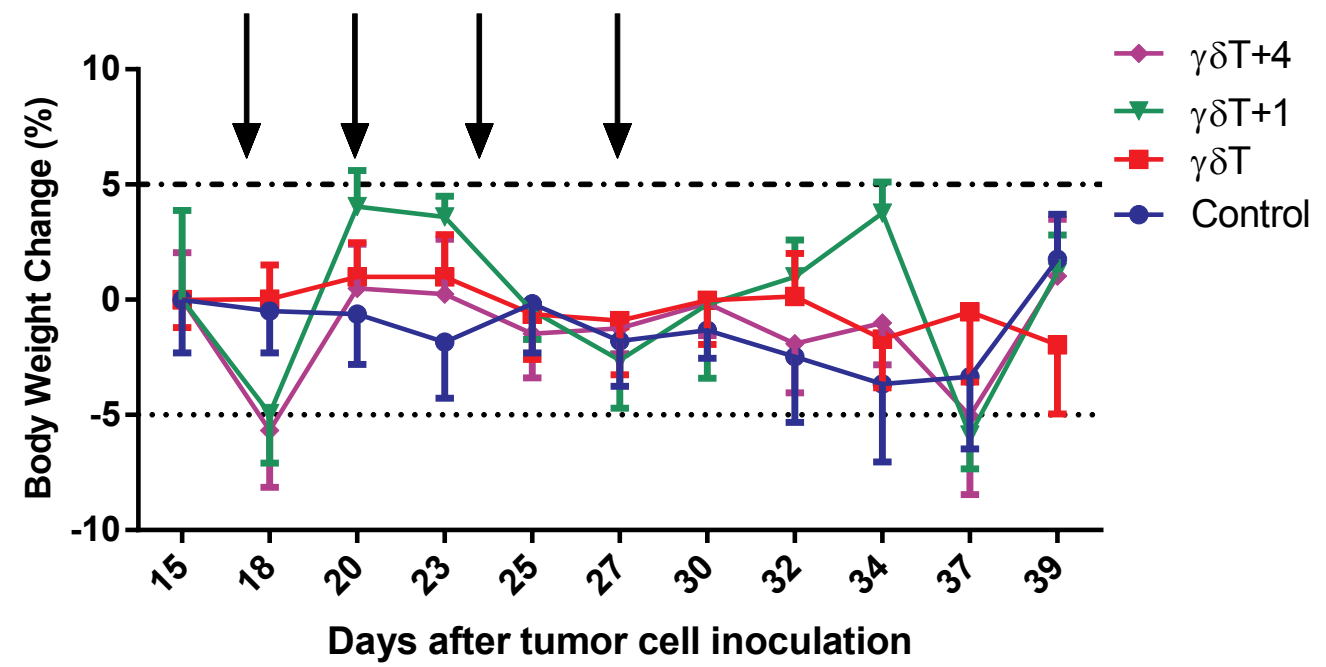

Sup.Fig.9

Supplement: Supplementary Figure 9 — The combination usage of the transfused Vγ2Vδ2 T cells together with Y111 inhibited significantly the tumor growth in vivo. (A) Curves of tumor burden for the individual mouse from the control group against the Vγ2Vδ2 T cells only group, the Vγ2Vδ2 T cells plus 1 mg/kg of Y111 group, or the Vγ2Vδ2 T cells plus 4 mg/kg of Y111 group. (B) Curves of body weight changes for each group. The black arrows indicated the treatment time point. Data are the mean ± SEM with 7 mice per group. [file DataSheet_9.pdf]
