# Supplementary material for: Bispecific Antibody PD-L1 x CD3 Boosts the Anti-Tumor Potency of the Expanded Vγ2Vδ2 T Cells
Source: Front Immunol. 2021 May 10;12:654080. doi: 10.3389/fimmu.2021.654080 (PMC8141752; doi:10.3389/fimmu.2021.654080)
Supplement: Supplementary Table 1 — The negative trend between Y111-induced killing ability (EC50) and PD-L1 positive percentages of tumor cell lines. a. The Pearson’s r and p-value was calculated as rMFI and EC50, PD-L1 positive percentages and EC50. [file Table_1.pdf]

	A549	H1299	H358	H1975	Pearson's r [a]	P value [a]
rMFI (MFI_{PD-L1}/MFI_{ISO})	3.07	3.74	6.5	26.11	-0.35	0.63119
PD-L1 percentages(%)	23.9	34	91.9	93.6	-0.54	0.43142
Y111-induced EC_{50} (pM)	24.3	1.8	5.7	4.7		

	A549	H358	H1975	Pearson's r [a]	P value [a]
rMFI (MFI_{PD-L1}/MFI_{ISO})	3.07	6.5	26.11	-0.65	0.455570471
PD-L1 percentages(%)	23.9	91.9	93.6	-1	3.01774E-05
Y111-induced EC_{50} (pM)	24.3	5.7	4.7		

Suppl Table 1. The negative trend between Y111-induced killing ability (EC_{50}) and PD-L1 positive percentages of tumor cell lines.

a. The Pearson's r and p-value was calculated as rMFI and EC_{50}, PD-L1 positive percentages and EC_{50}.
